# Investigating the Detachment of Glazed Ceramic Tiles Used in Buildings: A Brazilian Case Study

**DOI:** 10.3390/ma18020465

**Published:** 2025-01-20

**Authors:** Renato Freua Sahade, Priscila R. M. Leal, Sérgio S. Lima, Paulo Sérgio da Silva, Carlos R. C. Lima

**Affiliations:** 1 School of Engineering, Mackenzie Presbyterian University, São Paulo 01302-907, Brazil; carlos.lima@mackenzie.br; 2 Institute for Technological Research of the State of São Paulo, São Paulo 05508-901, Brazil; prileal@ipt.br (P.R.M.L.); sergiolima@ipt.br (S.S.L.); 3 Planville Construction Ltd., Curitiba 80035-230, Brazil; planville@gmail.com

**Keywords:** ceramic cladding, detachment, SEM, moisture expansion, XRF

## Abstract

Ceramic detachments in cladding systems are indicative of adhesion loss between the ceramic tiles and the substrate or its adhesive mortar due to inadequate quality workmanship, the quality of the adhesive mortar or that of the ceramic material, whether acting simultaneously or not. The shear stresses resulting from the ceramic tiles’ expansion due to humidity accelerate this process. There is a shortage of studies on the quality of ceramic tiles and adhesive mortars. This study conducted elemental, physical and microstructural characterization tests on ceramic tiles and adhesive mortars that showed detachment up to two years after being laid. At first glance, the adhesive mortar samples had adequate traits and degree of hydration. The ceramic tiles, on the other hand, showed high porosity and high levels of amorphous and poorly sintered materials, with no crystalline phase. In a second analysis, scanning electron microscopy (SEM) tests associated with boiling plus autoclave moisture expansion tests executed on unused ceramic pieces of the same conformation proved to be more suitable for predicting expansion potential than standard tests. Due to the costs and difficulties in accessing and analyzing the SEM tests, chemical analysis of the ceramic tiles was executed using X-ray fluorescence (XRF) to assess the presence of the amorphous silica (free quartz) and alkaline oxides. Together with pressure and temperature determination tests (autoclave), they may represent another alternative that is easier to access and more cost-effective for predicting future expansion.

## 1. Introduction

In the ceramic tile and sanitary ware market, Brazil has one of the world’s largest ceramic parks. According to the projections for 2024, Brazil stands out as the third-largest producer (808.2 million m^2^) and consumer (715 million m^2^) and is sixth in the export ranking. It produces some of the greenest ceramics in the world, with the lowest consumption of gas, energy and water per square meter. Ninety-eight percent of the country’s production is certified by a quality program. Regarding this, 72% is related to the production of ceramic tiles using the dry-pressed mass process, a process that enables products with lower costs and environmental impact in production [1].

Nevertheless, the product has had a history of technical non-conformities systematically occurring in Brazil since the early 1980s. Pathological manifestations associated with red-based ceramic tiles have been the subject of evaluation and study, as their frequency of occurrence in this type of tile has become a major concern for the construction industry and a recurring topic of research, discussed in various forums [2,3,4,5,6]. Studies point to a downward trend in their use both as coatings for internal use in buildings and for facade cladding [7].

Among the various problems that affect the performance of ceramic tiles, the detachment of ceramic tiles stands as one of the main issues due to the likelihood of accidents involving users and the cost of repair [8,9]. Detachments are characterized by the loss of adhesion of the ceramic tiles to the substrate or the adhesive mortar due to the inferior quality of the surface or adhesive, or the poor quality of the ceramic material, whether acting simultaneously or not [10,11,12,13,14].

**Figure 1:**
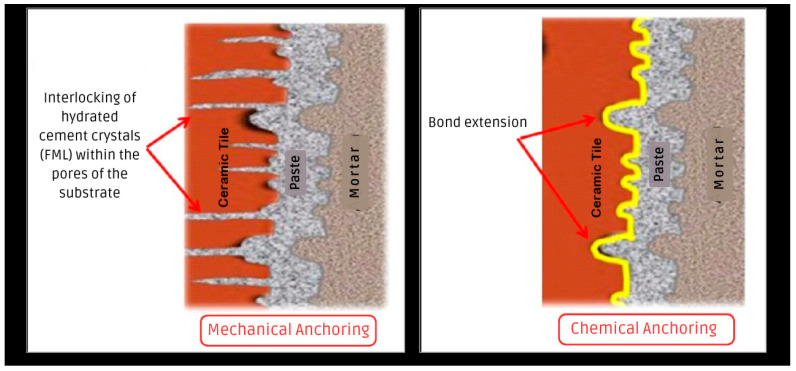
Schematic cross section of the adhesion mechanisms between ceramic tiles and adhesive mortars. Adapted from [15,16,17].

The adhesion mechanism between the ceramic tiles and the adhesive mortars operates both by mechanical anchoring, through the interlocking of crystals of the hydrated cement products of the adhesive mortar in the pores of the ceramic tiles (adherence strength), and by adherence extension (chemical anchoring or bond extension), as shown in Figure 1 [15,16,17,18].

The mechanisms that lead to the ceramic tiles’ detachment from the adhesive mortars are linked to the tensions generated at this interface when these tensions exceed the adhesion capacity between them [18]. One of the main phenomena leading to an increase in tangential stresses and the detachment of ceramic tiles is irreversible hygroscopic expansion due to humidity, known as moisture expansion (ME) [10,12,19].

**Figure 2:**
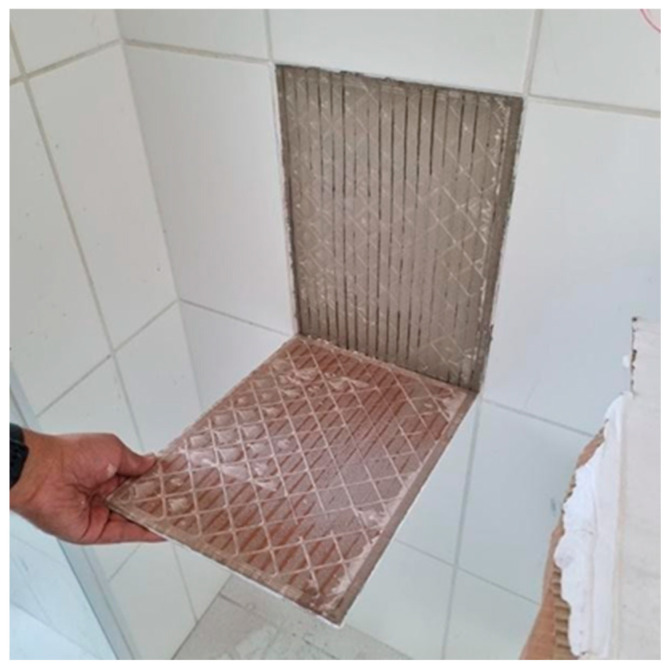
Detail of the ceramic tile detached from the adhesive mortar.

The ceramic tiles for internal use in a house development containing 448 housing units, located in the city of Jaraguá do Sul (SC), southern region of Brazil, showed widespread detachment in practically all these places. More than 13,000 m^2^ have peeled off without leaving any residue of adhesive mortar on the back of the ceramic tiles, as shown in Figure 2.

For the evaluation of the moisture expansion of ceramic tiles, they are boiled for 24 h, according the standards set out in [20]; however, this methodology shows moisture expansion results that are lower than the expansion results found in nature, according to recent studies [8,14].

The first sign that is indicative of detachment is the occurrence of a dull sound in the ceramic tiles when struck or the occurrence of puffing or bulging of the finishing layer (ceramic tiles and grouting), followed by the detachment of these areas, which may or may not be sudden. Unlike cementitious materials, some ceramic materials expand as they age as a result of the chemical reaction with water in the atmosphere [19,20].

ME is an adsorption process, where the rehydration reaction is controlled by the diffusion of water molecules in the surface’s open pores, which can adsorb moisture. This occurrence is correlated with the energy and surface area, characteristics of the glass phases, and is dependent on the chemical composition and quantity of alkali-containing glass phases defined in the firing phase [10,19,21,22,23,24]. ME is highly dependent on the raw materials and the fluxing materials [10,24,25,26]. Schurecht [27] first suggested that long-term moisture expansion is one of the causes of delayed cracking of glazed ceramic tiles, which was confirmed in later studies [10].

The addition of quartz tends to alter the pore structure of the material, as shown by the ratio Al_2_O_3_/SiO_2_. The lower this ratio, the greater the penetration of moisture into the piece and the easier it is for ME to take place. The higher the alkali content, such as Na_2_O and K_2_O in relation to the alumina, the greater the ME. Both conclusions have been demonstrated for ceramic materials with absorption of around 8% [28].

There is a significant reduction in ME with ceramic tile formulations that add calcite (CaCO_3_) at levels of up to 15%. The reason for the reduction is the elimination of the amorphous phase, where the clay material has reacted with the CaO during heating, giving rise to more crystalline calcium phases, such as anorthite (CaO.Al_2_O_3_.2SiO_2_), gehlenite (2CaO.Al_2_O_3_.SiO_2_), and wollastonite (CaO.SiO_2_) [29]. However, above 15%, the ME increases again since they lead the microstructure to excessive formation of the gehlenite phase (up to 6% by weight), which is responsible for a substantial increase in expansion due to humidity, since gehlenite combined with humidity increases the volume of the ceramic body and destroys its crystalline structure [30].

Higher ME values were observed in ceramic masses rich in well-crystallized kaolinite when fired at 1000 °C due to the formation of an aluminum and silicon spinel [25]. The transformation of the spinel into mullite, with more release of amorphous silica, occurred in the temperature range between 1050 °C and 1100 °C [31]. With the continuous increase in temperature, above 1050 °C the glass phase content increases even more, with a decrease in specific area and porosity, while the amorphous phases formed previously are transformed into crystalline phases, such as mullite (3Al_2_O_3_.2SiO_2_), anorthite (CaO.Al_2_O_3_.2SiO_2_), gehlenite (2CaO.Al_2_O_3_.SiO_2_), wollastonite (CaO.SiO_2_) and others, allowing the effect of reducing porosity to cause a reduction in ME [30].

In addition to the sintering temperature and the mineralogical composition of the raw materials that make up the ceramic mass, the sintering time is also important for completing all the physical and chemical reactions in the ceramic body. With the quick firing methods currently in practice due to production costs, large quantities of raw materials can remain unreacted, and the crystalline phases formed, as in the case of gehlenite, often remain in a semi-stable and intermediate form [30].

Long-term moisture expansions were often recorded in the range of 0.5 to 2.0 mm/m of linear deformation, a considerable deformation in the case of rigid construction systems and the cause of major stresses when material movements are restricted [20,31,32,33]. More conservative authors recommend that the limits be lower than 0.2 or 0.3 mm/m [8,31,32,33] or even as close to zero as possible [10].

The ME effect giving rise to pathology in ceramic tiles has therefore been the subject of intermittent studies over the last century, for both scientific and technological reasons [33], given that its occurrence brings financial and image losses to the ceramics and construction industry. Therefore, ME is responsible for cracking in the glaze [10,34,35], detachment of ceramic tiles when poorly adhered [10] or physical damage to restricted cladding systems when well adhered [19,21,32,36,37].

## 2. Materials and Methods

The materials used for this study were collected from a recent-built project in 2017, in the city of Jaraguá do Sul (SC), Brazil, presented previously. The test methodology for assessing the causes of the problems identified was divided into two stages. In the first stage, ceramic tiles were selected with a hollow sound under percussion; samples were then taken from the set formed by these glazed ceramic tiles and their respective adhesives, identified as detached ceramic tiles (DCTs—samples D1 to D5) and detached adhesive mortar (DAM—samples AM1 to AM5). Once these materials were characterized and the ceramic tiles were identified as not being completely sintered, which leads to high expansions due to humidity (moisture expansion (ME)), the second stage of tests focused on comparing unused ceramic tiles of the same type (UCTs—samples U1 to U5). The samples were taken both from the batch of detached pieces, identified as sample U2, as well as from four other manufacturers, identified as U1, U3, U4 and U5.

All the adhesive mortars for the tile samples were classified according to the ABNT NBR 14,081 standard [38] as type AC II (Brazilian adhesive mortar) or type C1 (according to DIN EN 12004:2007+A1:2012), the specifications of which are described in Table 1. The detached (D) and unused (U) ceramic tiles were classified according to ABNT NBR ISO 13,006 [39], whose specifications are described in Table 2.

The detached ceramic tiles (DCTs) and adhesive mortars (AMs) were characterized using thermal, mineralogical, chemical and physical analyses.

For differential thermal analysis (DTA) and simultaneous thermogravimetric analysis (TGA) of both materials, the general guidelines of ASTM E 794-06 (2012) [40] were used. The equipment used was TA Instruments, model SDT 2960, where the material was ground in a ring mill until it passed completely through the ABNT sieve n°. 325 (0.045 mm) and an alumina crucible without a lid, with a gas flow of 50 mL/min of nitrogen (N_2_) and a heating rate of 10 °C/min up to 1000 °C.

For the mineralogical analysis of DCTs by X-ray diffraction (XRD), a Malvern Panalytical (Almelo-Netherlands) diffractometer, model Empyrean, was used, using a 1/4° divergent slit, 1/2° anti-scatter slit, operating at copper Kα radiation with 45 kV-40 mA, scanning at 2°/min and ranging from 3° to 30°. The identification of the compounds was performed using the X-pert HighScore Plus software (version 3.0) from Panalytical, and diffractometric patterns were provided by the ICDD (International Center for Diffraction Data) with updates up to 2003. The diffractograms obtained were interpreted according to the JCPDS ICDD mineral sheets (Joint Committee on Powder Diffraction Standards, 1974). Selected powder diffraction data for minerals, Databook, Swarthmore, 833p. The areas of the most intense peaks of each mineral identified in the ceramic material were used for semi-quantification.

For the mineralogical analysis of AM by X-ray diffraction (XRD), a Malvern Panalytical (Almelo-Netherlands) diffractometer, model Empyrean, was used, with a Pixel3D detector, operating in copper Kα radiation with 45 kV-40 mA and scanning of 0.02°/min, following the general guidelines of ASTM C1365-18 [41]. Compound identification was performed using the X-pert HighScore Plus software (version 4.5 (4.5.0.22741)) from Panalytical, and diffractometric and structural patterns were provided by the free COD (Crystallography Open Database-updated in 2016) and, eventually, diffractometric and structural patterns from the ICDD (International Center for Diffraction Data) and ICSD (International Center for Structure Data), respectively. The statistical indicator used to verify the refinement results was the GOF (goodness of fit), in addition to the graph of the differences between the observed and calculated diffractograms. Below are some of the refinement parameters used in each diffractogram to obtain the percentages of the phases present, in respective order of application: refinement of the scale factor, adjustment of the baseline, refinement of the diffractometer constant (Zero Shift), refinement of the unit cell of the larger phases, refinement of the unit cell of the smaller phases, refinement of the peak profile (for phases present with more than 5%) and refinement of the preferred orientation (for susceptible compounds).

For the mineralogical analyses by X-ray diffraction (XRD), both materials were ground in a ring mill until they passed completely through the ABNT N°. 200 sieve (0.075 mm).

For the chemical analysis of the DCTs, a Malvern Panalytical (Almelo-Netherlands) X-ray fluorescence spectrometer (XRF), model Minipal Cement, was used, from pellets melted in a Claisse model M4 melting machine, using fluxes based on a mixture of lithium tetraborate/lithium metaborate, brand MAXXIFLUX (São Paulo-Brazil) (66.67% Li_2_B_4_O_7_, 32.83% LiBO_2_ and 0.70% LiBr), with a proportion of 0.6 g of sample and 6.75 g of flux. Using castings of the materials fragmented with the aid of a hammer, they were crushed and ground in a vibratory Disc Mill RS 200 (Haan, Germany) until they passed completely through an ABNT N°. 20 (0.84 mm) sieve. The results were estimated by semi-quantitative analysis based on the general guidelines of ABNT NBR ISO 12,677 [42]. For the AM, the chemical analysis was obtained by the mix proportion reconstitution method based on the general guidelines of the test method presented in the work by Quarcioni and Cincotto [43].

From the same portion that the sample was extracted from for petrographic analysis, we focused on the portion of the ceramic body, without analyzing the engobe and glaze, in the position perpendicular to the bedding of the ceramic piece, covering this fragment with a thin layer of gold to promote conductivity. The microstructure of the ceramic plates was investigated using an FEI Quanta 400 (Hillsboro-EUA) field emission scanning electron microscope (SEM) under accelerating voltage conditions of 15 to 20 kV and a working distance (WD) of 10 mm.

The ceramic tiles were also characterized for water absorption (*E_v_*), moisture expansion (ME) and crazing resistance using the test methods prescribed by the ABNT NBR ISO 10,545 standards, parts 3, 10 and 11, respectively [20,44,45].

The vacuum method was referenced for the determination of water absorption, according the ABNT NBR ISO 10,545 standard, part 3 [44]. This method was applicable only to classification of ceramic tiles. The samples were dried to constant mass in an oven capable of operating at 110 ± 5 °C for 24 h. They were then cooled in a desiccator over silica gel, using a balance with an accuracy of 0.01% to weigh the sample mass ($m_{1}$). The samples were then placed vertically in the vacuum chamber up to a pressure of 10 ± 5 kPa and held for 30 ± 2 min. Then, while maintaining the vacuum, enough water was added to cover the plates by at least 5 cm. After releasing the vacuum and allowing the plates to remain submerged for 15 ± 2 min, the samples were lightly dried with a microfiber cloth, and the saturated mass ($m_{2})$ of each sample was determined to the nearest 0.01% of the mass. For each plate, the water absorption, $E_{v}$, expressed as the percentage of dry mass, is calculated using Equation (1):

(1) $$Ev=100\times \frac{\left(m_{2}-m_{1}\right)}{m_{1}}$$

For the evaluation of the moisture expansion of ceramic tiles (ME), two methods were used: boiling for 24 h [20] and autoclave. Whole tile samples were cut from the center of each tile and refired in a kiln with a temperature rise rate of 150 °C/h and a 2 h step at 550 ± 15 °C. The samples were cooled inside the kiln until reaching 70 ± 10 °C and were then kept at room temperature for 24 h in a dry desiccator. The first measurement, that’s the re-firing shrinkage of the ceramic tile (RS), i.e., is the difference between the dimension measurement before and after the re-firing. The boiling method consists of immersing the samples of ceramic tiles in deionized or distilled water after determining the RS, where they boil for 24 h. The ME value, determined according to said standard, is the specific increase in length that the specimen undergoes after spending 24 h in boiling water, taking as reference the RS and its cooling. On the other hand the autoclave moisture expansion test, which complements the standard 24 h boiling test, consists of the specific increase in length that the specimen undergoes after remaining in boiling water for 24 h, followed by 5 h in an autoclave under a pressure of 500 ± 20 kPa and a temperature of 159 ± 1 °C, taking its length after reheating and cooling as a reference (refiring shrinkage).

For the evaluation of the crazing resistance [45], the samples, obtained from the DCTs and UCTs, are subjected to the application of methylene blue solution to identify whether cracking is present or not, and this is recorded. If no cracking is observed, the samples are refired in a kiln with a temperature rise rate of 150 °C/h and a 2 h step at 550 ±15 °C, cooled to room temperature and subjected to the application of methylene blue solution again, to verify whether cracking is present or not, and this is recorded. If no cracking is observed, the test specimens are autoclaved for 2 h under a pressure of 500 ± 20 kPa and then subjected to the application of methylene blue solution again to verify whether cracking is present or not, and this is recorded.

The tests executed, the number of samples tested and their identification are shown in Table 3.

As shown in Table 3, only absorption, moisture expansion, crazing resistance and SEM tests with chemical analysis by energy dispersive spectroscopy (EDS) were carried out on the unused ceramic tiles (UCTs) in order to investigate the microstructure and compare them with the detached ceramic tiles (DCTs).

## 3. Results

### 3.1. Characterization of Ceramic Tiles

#### 3.1.1. Determination of Water Absorption ($E_{v}$)

Table 4 shows the results of the water absorption tests for the detached (D1 to D5) and unused (U1 to U5) ceramic tiles.

#### 3.1.2. Determination of Moisture Expansion (ME)

**Figure 3:**
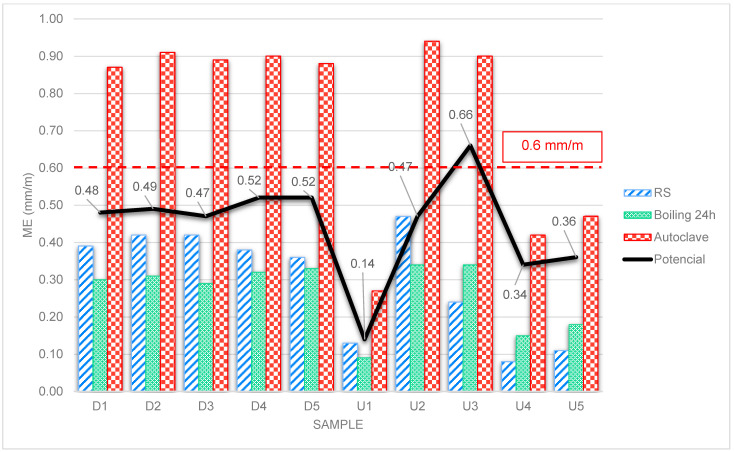
Graph of the moisture expansion test results for the detached (D) and unused (U) ceramic tile samples.

Figure 3 shows the results of the determination of moisture expansion by refiring shrinkage (RS), by boiling for 24 h and by autoclave for both the detached samples (D1 to D5) and the unused samples (U1 to U5).

The dashed red line in Figure 3 refers to the limit suggested by the ABNT NBR ISO 10,545 part 10 standard of 0.06% or 0.6 mm/m [20]. The black line refers to the potential growth that the ceramic tiles could undergo compared to the autoclave and RS tests, since the autoclave test is used to accelerate the ME of ceramic pieces by means of pressure and temperature and thus provide a growth prediction.

#### 3.1.3. Determination of Crazing Resistance

Table 5 shows the results for determination of crazing resistance after autoclaving of detached (D1 to D5) and unused (U1 to U5) ceramic tiles.

#### 3.1.4. Thermogravimetric Analysis Results (TGA/DTA)

The results of the thermogravimetric analysis of the detached ceramic tiles (DCTs) are shown in Table 6, and the interpretation of the mass losses as a function of the temperature range for the detached ceramic tiles is shown in Table 7. Supplementary File S1 contains the original curves for each sample of the DCTs.

#### 3.1.5. X-Ray Diffraction (XRD) for DCT

**Figure 4:**
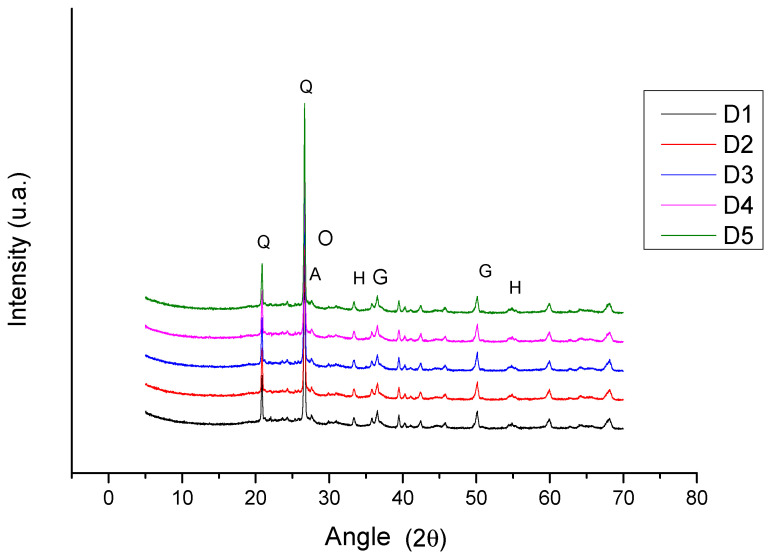
X-ray diffractogram for the detached samples D1 to D5 (Q—quartz; H—hematite; G—goethite; A—albite; O—orthoclase). The amorphous halo can be seen in the region between 18 and 29° 2θ.

The results of the diffractometric analysis on the detached ceramic tiles are shown in the graph in Figure 4 for samples D1 to D5. Supplementary File S2 contains the original curves for each sample of the DCTs.

#### 3.1.6. Semi-Quantitative Analysis by X-Ray Fluorescence (XRF)

The results obtained from the semi-quantitative XRF analyses are shown in Table 8.

#### 3.1.7. Scanning Electron Microscopy of the Ceramic Plates (SEM)

**Figure 5:**
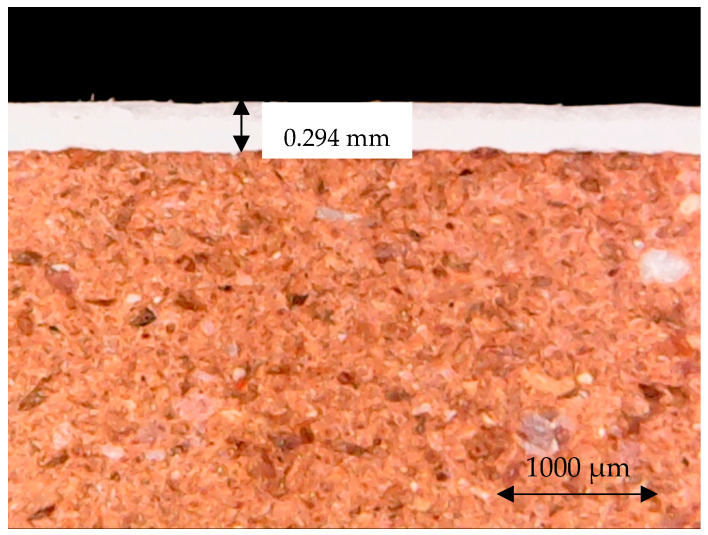
Fracture cross section of the ceramic tiles studied from the DCT and UCT samples (mag. 1000×).

In the scanning electron microscopy of the fracture surfaces (Figure 5) of the ceramic tiles studied (DCT and UCT samples), the occurrence of phyllosilicates was investigated, as their presence is not desirable in ceramics calcined at temperatures above 900 °C.

**Figure 6:**
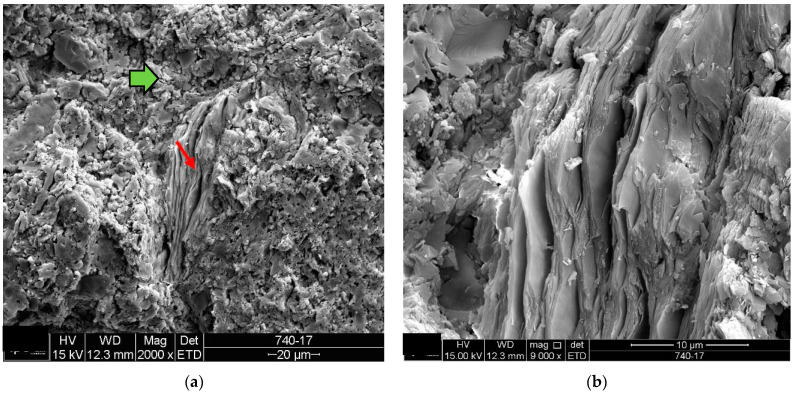
Sample D1: (**a**) Fragment of phyllosilicate (red arrow) in the middle of the porous mass (green arrow)–mag. 2000×; (**b**) detail of the phyllosilicate of image (**a**)-mag. 9000×. Note the preserved foliated appearance. Secondary electrons.

**Figure 7:**
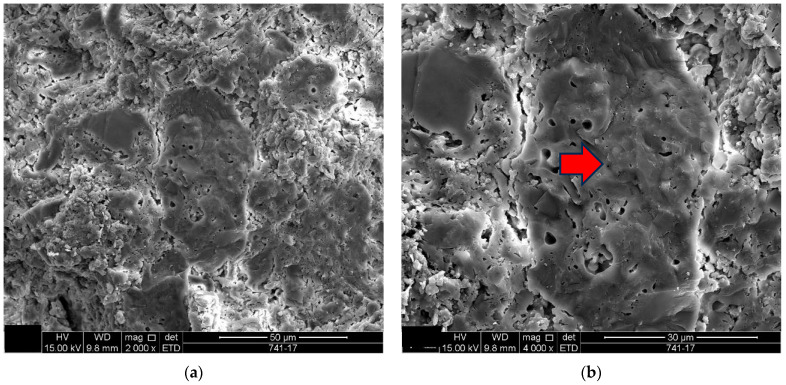
Sample D2: (**a**) Vitreous mass clump in the middle of the porous mass-mag. 2000×; (**b**) the red arrow indicates the clump of vitreous mass of image (**a**) in a surrounding porous mass with highlighted edges-mag. 4000×. Secondary electrons.

**Figure 8:**
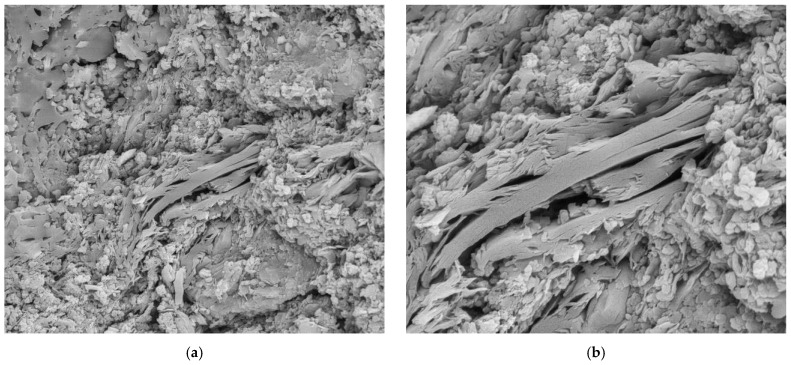
Sample D3: (**a**) Fragments of phyllosilicates with preserved foliation in the middle of the porous mass-mag. 1000×); (**b**) detail of the phyllosilicate of image (**a**)-mag. 4000×. Secondary electrons.

**Figure 9:**
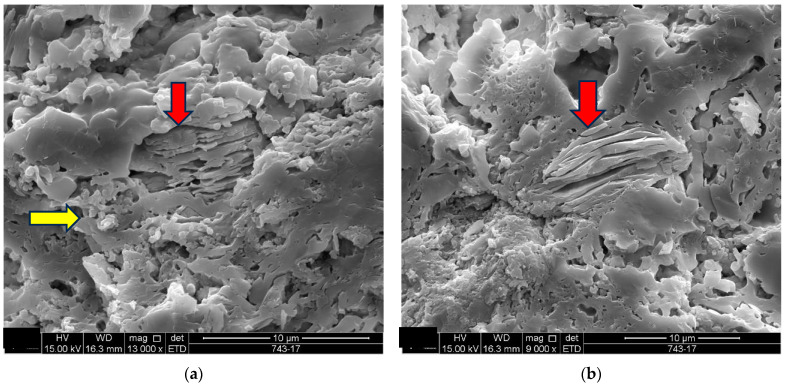
Sample D4: (**a**) The red arrow indicates partially fused phyllosilicate with preservation of the foliated microstructure, and the yellow arrow indicates the surrounding porous mass-mag. 13,000×; (**b**) the red arrow indicates partially fused phyllosilicate with preservation of the foliated microstructure-mag. 9000×. Secondary electrons.

**Figure 10:**
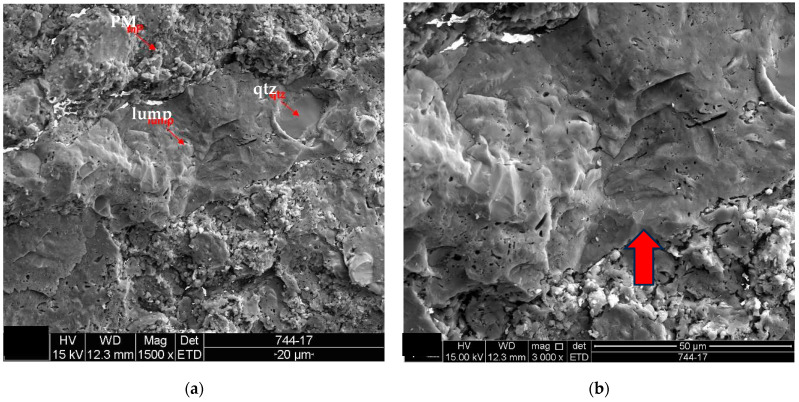
Sample D5: (**a**) Porous mass (PM) arranging itself as a matrix for lump with quartz fragment included (qtz)-mag. 1500×; (**b**) the red arrow indicates the clump of vitreous mass in image (**a**) in a surrounding porous mass with highlighted edges-mag. 3000×. Secondary electrons.

The analysis results for the detached tiles (D1 to D5) are shown in the electron micrographs in Figure 6, Figure 7, Figure 8, Figure 9 and Figure 10.

**Figure 11:**
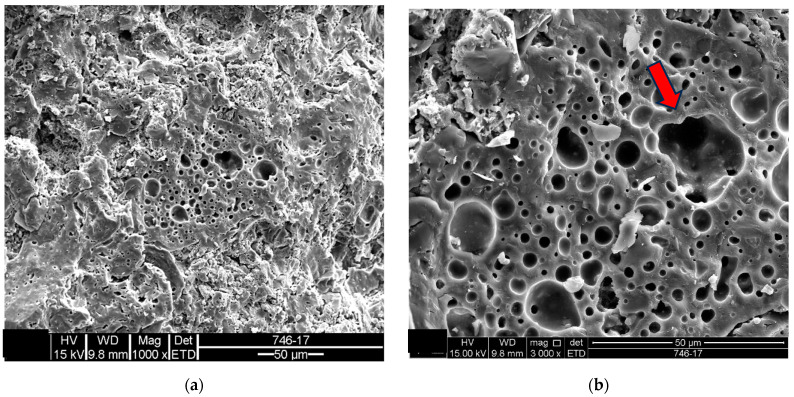
Sample U1: (**a**) Vitreous mass core in the center-mag. 1000×; (**b**) detail of the vitreous mass core from the micrograph in (**a**)-mag. 3000×. The red arrow indicates the EDS microanalysis in Figure 12. Secondary electrons.

**Figure 12:**
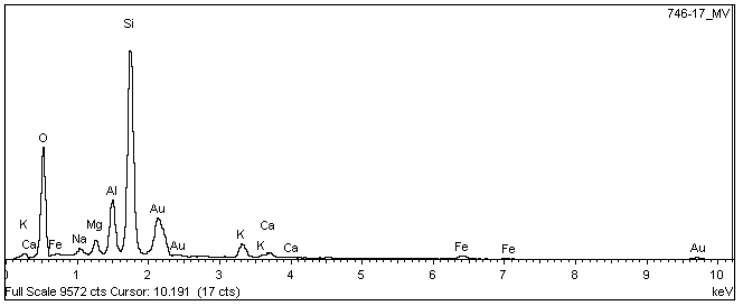
EDS microanalysis of the vitreous mass core in Figure 11b. Full scale 9572 cts. Cursor: 10,191 (17 cts).

**Figure 13:**
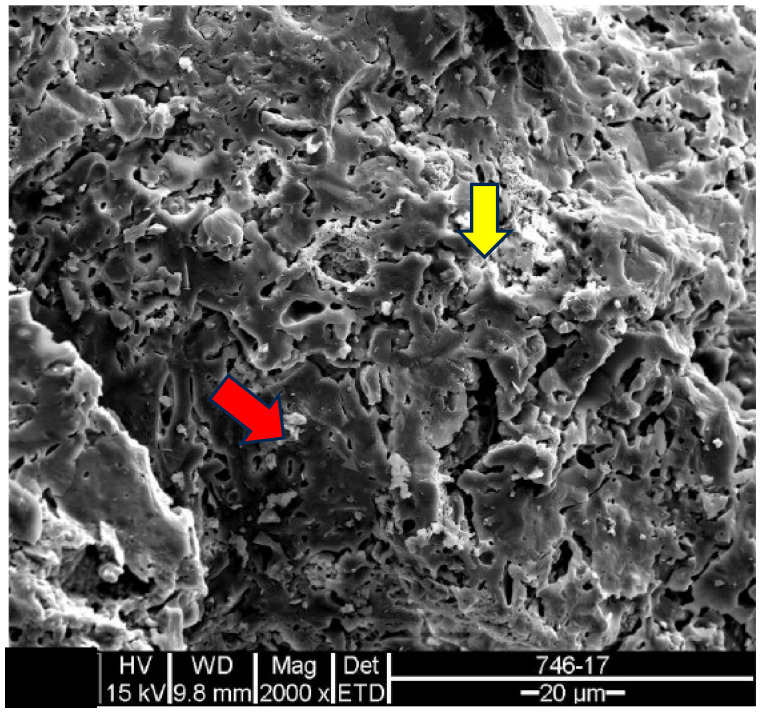
Sample U1: Porous mass (red arrow) with detail of the presence of aluminous silica phase (yellow arrow)–mag. 2000×. Secondary electrons.

**Figure 14:**
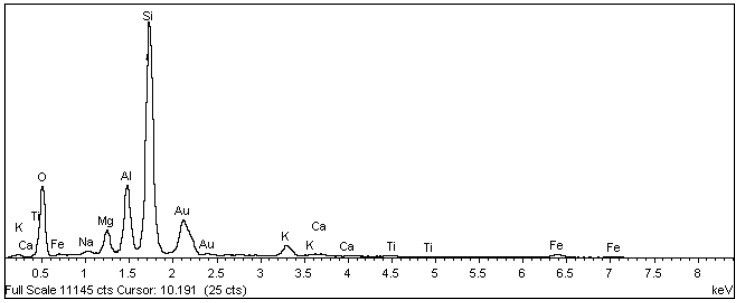
EDS microanalysis of the point indicated by the red arrow for sample U1: porous mass (Figure 13). Full scale 11,145 cts. Cursor: 10,191 (25 cts).

**Figure 15:**
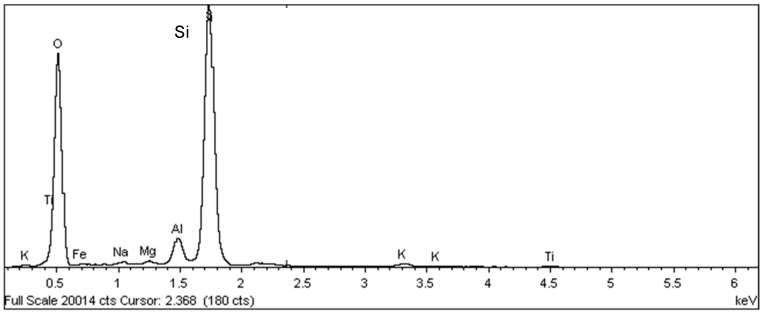
EDS microanalysis of the point indicated by the yellow arrow for sample U1: detail of the presence of aluminous silica phase (Figure 13). Full scale 20,014 cts. Cursor: 2368 (180 cts).

**Figure 16:**
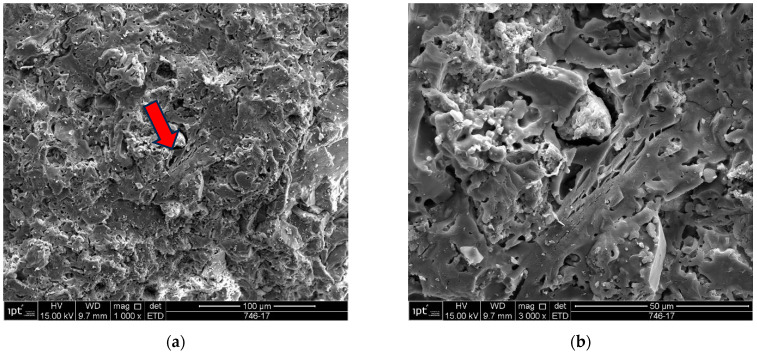
Sample U1: (**a**) Porous mass with details of the presence of phyllosilicate-mag. 1000×; (**b**) detail of the porous mass and phyllosilicate from the micrograph at the red arrow in (**a**)-mag. 3000×. Note that this “phyllosilicate” is already collapsing, i.e., partially fused. Secondary electrons.

**Figure 17:**
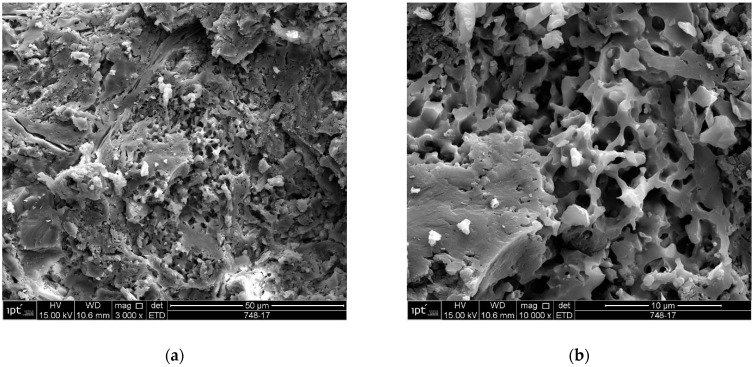
Sample U3: (**a**) Fracture surface with porous mass-mag. 3000×; (**b**) detail of the porous mass in the micrograph in (**a**)-mag. 10,000×. Secondary electrons.

**Figure 18:**
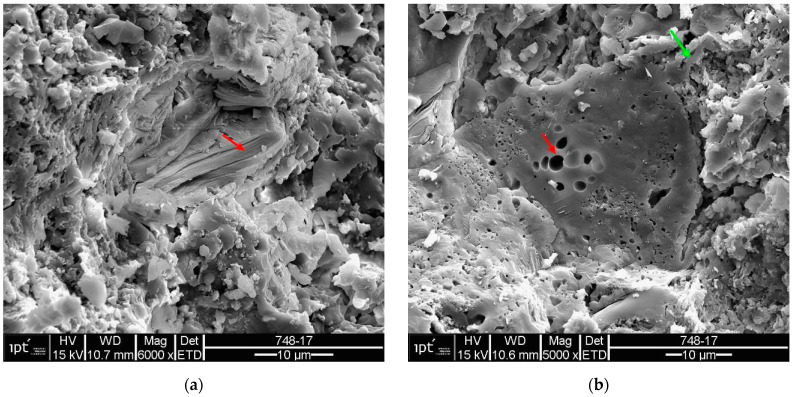
Sample U3: (**a**) Electron micrograph showing phyllosilicate in porous mass (red arrow)-mag. 6000×; (**b**) the electron micrograph shows the vitreous mass (red arrow) and surrounding porous mass (green arrow)-mag. 5000×. Secondary electrons.

**Figure 19:**
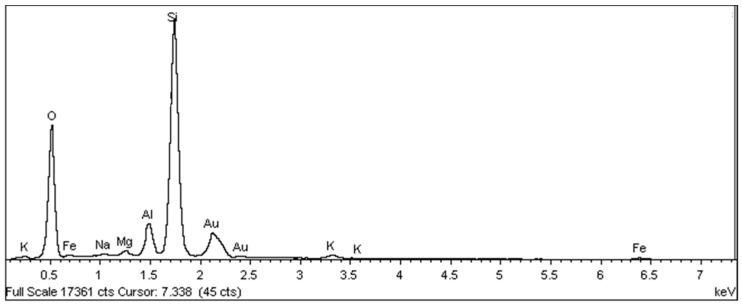
Sample U3: EDS microanalysis indicated by the red arrow in Figure 18a: phyllosilicate in porous mass. Full scale 17,361 cts. Cursor: 7338 (45 cts).

**Figure 20:**
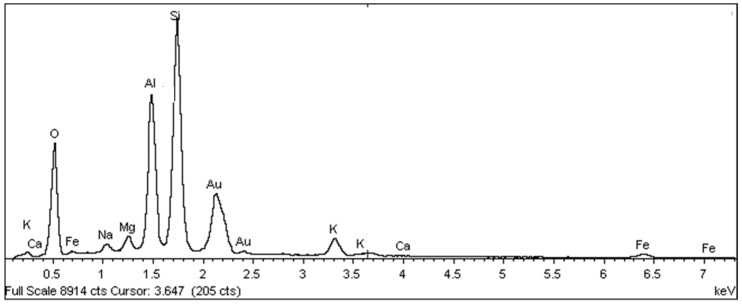
Sample U3: EDS microanalysis spots of the vitreous mass indicated by the red arrow in Figure 18b. Full scale 8914 cts. Cursor: 3647 (205 cts).

**Figure 21:**
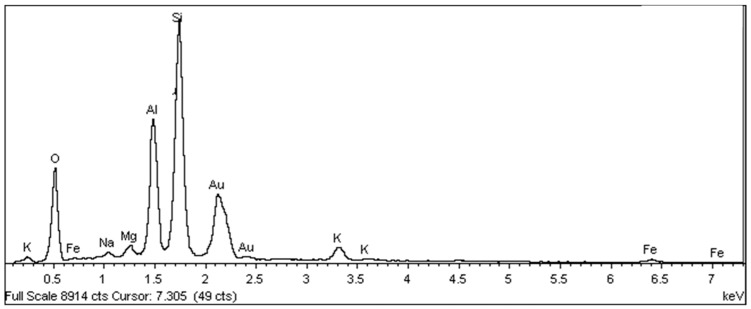
Sample U3: EDS microanalysis spots of the surrounding porous mass (green arrow) in Figure 18b. Full scale 8914 cts. Cursor: 7305 (49 cts).

**Figure 22:**
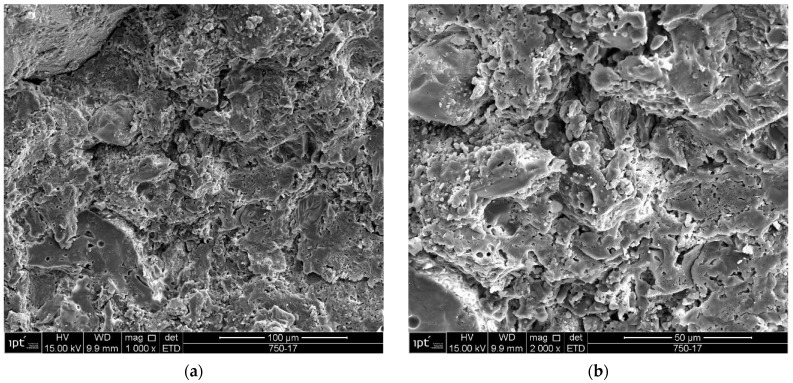
Sample U5: (**a**) Porous mass predominant in the sample-mag. 1000×; (**b**) detail of the porous mass from the electron micrograph in (**a**)-mag. 2000×. Secondary electrons.

Samples of the unused ceramic tiles U1, U3 and U5 were taken for analysis by SEM in order to obtain parameters for comparison with the five samples of detached ceramic tiles (DCT). Figure 11, Figure 12, Figure 13, Figure 14, Figure 15, Figure 16, Figure 17, Figure 18, Figure 19, Figure 20, Figure 21 and Figure 22 show the results of the electron micrographs of the unused ceramic tile (UCT) samples and the results of the chemical characterization by X-ray scattering (EDS) of these samples. Supplementary File S5 contains the original curves EDS for each sample of the UCT.

### 3.2. Characterization of Adhesive Mortars

#### 3.2.1. Thermogravimetric Analysis (TGA/DTA)

The results of the thermogravimetric analysis of the adhesive mortars used to fix the detached ceramic tiles (DAM) are shown in Table 9. The interpretation of the mass losses as a function of the temperature range for the mortars is shown in Table 10. In Supplementary File S3, it is possible to see the curves of the TGA/DTA analysis for all samples.

#### 3.2.2. X-Ray Diffraction (XRD) for Adhesive Mortars

**Figure 23:**
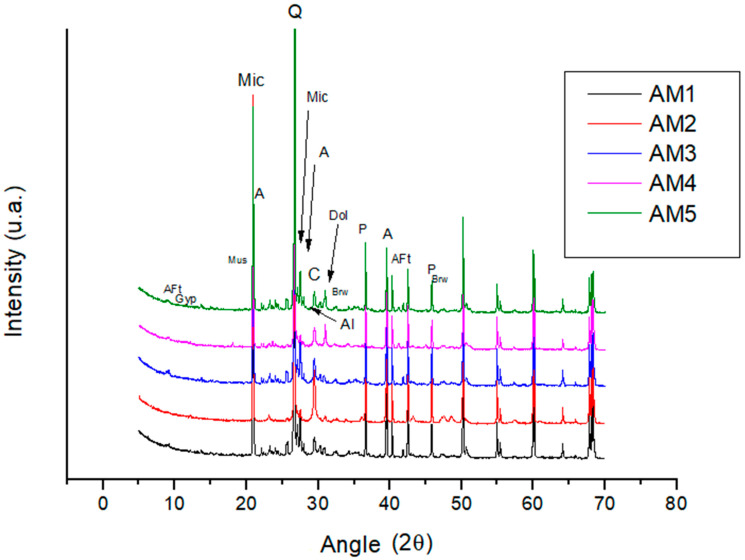
X-ray diffractograms of samples AM1 to AM5 (Q—quartz; Mic—microcline; A—albite; Mus—muscovite; C—calcite; P—portlandite; Gyp—gypsum; Brw—brownmillerite; AFt—ettringite; Al—alite (C_3_S); Dol—dolomite).

The results of the diffractometric analysis for the mortars used to fix the detached ceramic tiles (DAM) are shown in Figure 23 and Table 11. In the mineralogical analysis, the minerals identified as quartz, feldspar (microcline), albite, muscovite (mica), calcite and dolomite come from the aggregate fraction and/or mineral additions typical of Portland cement. With the exception of sample AM2, the other samples show a slight presence of non-hydrated calcium silicates (allite [C_3_S] and/or belite [C_2_S]) from the binder material, which also contributes to the presence of brownmillerite, portlandite and ettringite (Aft), indicating that the cement (binder) is hydrated. In Supplementary File S4, it is possible to see the curves of XRD analysis for all samples.

#### 3.2.3. Chemical Analysis of Adhesive Mortars

The results obtained from the chemical analyses expressed on an original basis and on a non-volatile basis are shown in Table 12.

#### 3.2.4. Proportion Mix Reconstruction

With the values obtained from the samples in the non-volatile base (Table 12), the contents of the constituents present were calculated, and the respective proportion mix of the mortars tested was estimated (Table 13), adopting the following assumptions:

- The fire loss represents the combined water and CO_2_ present in the binder (cement).
- The insoluble residue in hydrochloric acid represents the content of siliceous aggregate (sand) and the solubilized fraction represents the binder composed of cement.
- The cement content was calculated based on the silicon anhydride content (SiO_2_) of the mortar, expressed on a non-volatile basis, and using a Portland cement type CP II-F (Table 14), according to the Brazilian standard, or CEM II/B-L, according to the European standard), as a reference parameter for samples AM2 and AM4, given the carbon dioxide content (CO_2_). For samples AM1, AM3 and AM5, Portland cement type CP II-E (Table 14), according to the Brazilian standard, or CEM II/A-S, according to the European standard, was adopted as the reference parameter.
- The cement content was recalculated on the original basis (Table 12), from the cements adopted as reference (Table 14) and on a 100% basis together with the aggregate content.

Considering that the maximum limit of sulfuric anhydride (SO_3_) specified for Brazilian Portland cement types CP I, CP II, CP III and CP IV is 4.0%, which are equivalent to Portland cements type CEM I, CEM II, CEM III, CEM IV and CEM V according to the European standard EN 197-1 [46], the respective limits of SO_3_ theoretically coming from the cement, were also calculated for five (05) types of simple cement mortar. Table 15 shows the SO_3_ limit values calculated in order to support a comparative assessment with the SO_3_ result, on a non-volatile basis shown in Table 12, as a function of the cement contents calculated (Table 13).

It should be noted that the presence of sulfide (S^2−^) indicates the use of cement containing blast furnace slag (BFS) as an addition in accordance with the respective technical specifications for CP I-S, CP II-E or CP III (according to the Brazilian standard) or CEM II/A-L, CEM II/A-S or CEM III (according to the European standard), respectively, and the absence of sulfide (S^2−^) indicates the use of cement without the addition of slag, such as CP I, CP II-Z, CP II-F, CP IV and CP V ARI-RS (according to the Brazilian standard) or CEM I, CEM II/A-P, CEM II/B-L, CEM IV and CEM V (according to the European standard), respectively.

## 4. Discussion

It can be seen in Table 4 that according to the ABNT NBR ISO 13,006 standard [39], the samples of tiles were classified as Group BIIb: dry-pressed ceramic tiles with a degree of absorption in the range 6% < *E_v_* ≤ 10%.

For all the DCT samples (D1 to D5), it can be seen that the ME by 24 h boiling test in Figure 3, which is prescribed in the standard, shows lower moisture expansion results than the results of the expansion from refiring shrinkage, i.e., nature showed greater effective growth compared to the standard test, confirming studies that show that, in ceramic pieces that have taken off, the standard test does not give reliable results in terms of growth potential, and that it is recommended to measure the difference between the piece before and after refiring (RS) [8,14].

In the moisture expansion test according to the normative recommendations, it was noted that the detached samples showed average results 21% lower than the average of the expansions that occurred by refiring shrinkage. The unused board representative of the detached batch (U2) showed expansion 52% higher than the average of the normative expansion results of the detached samples (DCTs). Compared to the refiring shrinkage and normative expansions of the U2 board (0.47 and 0.34 mm/m, respectively), representative of the detached samples, in relation to the unused samples (0.14 and 0.19 mm/m, respectively), the moisture expansion test showed 3 to ~2 times more expansion, respectively.

For the unused samples of the graph in Figure 3 (U1 to U5), it can be seen that, except for samples U1 and U2, the other samples showed higher ME due to boiling than the ME by RS. This can be explained by the fact that the pieces were recently fired (new). As boiling causes pores that were closed to open, the pore volume increases, and consequently, water absorption increases [25]. Thus, the ME test carried out on new parts is not significant [14].

When analyzing the ME in boiling for 24 h and in an autoclave, and the microstructure by SEM of samples U2 and U3, it can be seen that sample U3 is the most similar in its microscopic morphological arrangement to the detached samples (D1 to D5). From this observation, it can be inferred that the 24 h boiling test for new BIIb ceramic slabs is not sufficient to “predict” long-term ME, compared to the autoclave test.

Still, in the analysis of Figure 3, when comparing the samples of detached ceramic tiles (D1 to D5) with tiles from the same source unused (U1 to U5), U2 has higher values than ME. Therefore, it is inferred that the unused pieces are more exposed (surface area) to ambient humidity and, consequently, to greater adsorption and expansion than the pieces already in contact with the adhesive mortar.

When comparing the water absorption samples of DCTs and UCTs with ME, there is no correlation between them. This can be explained by the fact that ME is caused by a reduction in the surface tension of the individual solid phases within the ceramic bodies when moisture is adsorbed to their surface. When the surface tension is reduced by the chemical interaction (chemisorption) of the surface atoms, the particle expands [19,47].

According to ABNT NBR ISO 13,006 [39], regardless of the water absorption group to which the ceramic tiles belong, the requirement for crazing resistance is that the glaze does not crack. Moisture expansion controls the stability of the shape after installation, as well as the crazing of the glaze over time. The greater the moisture expansion, the more the body of the panel expands, developing tensile stresses on the glaze, resulting in cracks (crazing) [10,21,24].

Analysis of the graph in Figure 3 shows that samples U2 and U3 showed similar results for the 24 h boiling and autoclave ME tests, although sample U3 is the one that is most similar morphologically when analyzed by SEM to the plates in the detached group (DCTs). However, no crazing of the enamel was observed in sample U3. Thus, it can be said that ceramic pieces with cracked glaze are indicatives of high ME [10,36]; high moisture expansions are not necessarily responsible for the crazing of the glazed tiles, though the expected value for the expansion due to humidity of a ceramic coating depends on the dilatometric agreement between the thermal expansion of the glaze and the ceramic body [22]. Glazes must have a lower coefficient of thermal expansion than the ceramic body so that the glaze always remains under compression, and the expansion of the body due to humidity must not exceed the compressive forces of the glaze [10].

Thermogravimetric analysis (TGA) for all the samples of the DCTs, as shown in Table 6 and Table 7, indicated endothermic peaks between 25 °C and 150 °C corresponding to the loss of free water of 0.28% to 0.58% and loss of water adsorbed in the interlamellar space of the mineralogical phases, with dehydroxylation of goethite and residual clay minerals of 0.46% to 1.10% (between 150 °C and 850 °C). A slight endothermic reaction is observed between 400 °C and 800 °C, which indicates the possible transformation of either residual kaolinite or another phyllosilicate present, with the formation of an intermediate phase. Between 850 °C and 1100 °C, the exothermic peaks are related to the nucleation of mullite and cristobalite. It was noted that nearly all the samples showed melting when tested above 1100 °C.

Qualitative mineralogical analysis (XRD) of all the samples of the DCTs (D1 to D5) revealed the presence of quartz (SiO_2_), iron oxide (hematite—Fe_2_O_3_; goethite—FeO (OH)), feldspar (albite—Na.Al.Si_3_O_8_; orthoclase—K.Al.Si_3_O_8_) and amorphous material. The presence of the mullite crystalline phase was not detected. Semi-quantitative analysis by X-ray diffraction (XRD) showed high levels of non-crystallized phase (amorphous phase and/or glassy phase between 53.4% and 58.3%) and free quartz (between 37.7% and 40.7%).

The presence of high levels of amorphous and/or glassy phase is responsible for the high ME observed in the detached samples, shown in the graph in Figure 4, indicating that they were either fired at medium temperatures (between 950 °C and 1050 °C), temperatures at which the amorphous phase and porosity are more intense [25,28,48] or the sintering time was not enough to complete all the physical and chemical reactions in the ceramic body [30].

Analysis of the chemical composition results shown in Table 8 for DCTs are basically made up of silica (72.3%), Al_2_O_3_ (12.5%), Fe_2_O_3_ (5.7%), K_2_O (3.2%), Na_2_O (1.4%), MgO (1.2%), CaO (1%) and low levels of other elements (<1%). The ratios Al_2_O_3_/SiO_2_ of around 0.17 and (Na_2_O + K_2_O)/Al_2_O_3_ of around 0.37 indicate that the samples analyzed have a high potential for ME, compared to the samples analyzed by Young and Brownell in 1959 for pieces with an absorption level of around 8%, as is the case with samples D1 to D5, which had an average absorption of 7.5%, shown in Table 4 [28]. The high quartz content (SiO_2_) tends to alter the pore structure of the material (amorphous silica), allowing moisture to penetrate more easily, increasing the ME [24,48,49]. The ME increases with the presence of an alkaline glassy phase, mainly Na and K, and an amorphous phase containing alkaline oxides (Na_2_O and K_2_O) in the fired ceramic body [10], and generally decreases with an increase in the crystalline phases and the alkaline-earth content (Ca and Mg) of the structure [30].

The microstructure of the detached ceramic samples (D1 to D5) analyzed by SEM can be divided into three elements, as shown in Figure 6, Figure 7, Figure 8, Figure 9 and Figure 10:

(i) Fragments of phyllosilicates with totally and/or partially preserved foliation.
(ii) Porous mass arranging itself as a matrix.
(ii) Clumps of vitreous mass in abundance but isolated by the porous mass matrix. By observing the morphologies under an electron microscope, it can be interpreted that the porous mass, the most abundant in volume, is the result of the partial fusion of phyllosilicates, as it is often possible to see the preservation of the leafy microstructure (characteristic of phyllosilicates) making up the porous mass. The vitreous mass, on the other hand, is the morphological expression of the complete sintering of the raw materials.

The arrangement of phyllosilicate cores and vitreous mass clumps in a porous mass matrix can be hypothetically explained by the greater ease of localized fusion (expressed by the vitreous mass cores), facilitated by some material with a lower fusion temperature (flux). Another inference is that the heterogeneity of the raw materials (either from the mixture of phases or from the humidity) led to the microstructural arrangement observed with the firing temperature and/or time being insufficient for the homogenization of the ceramic piece through total sintering of the raw materials.

The microstructural analyses correspond to the results obtained in the thermal, diffractometric and chemical X-ray fluorescence analyses, since no crystalline structure (mullite and/or cristobalite) was detected in the detached samples. The presence of quartz is visible amidst the vitreous mass and porous matrix. The SEM analyses are also correlated to the physical characterization tests, such as the moisture expansion and the crazing resistance tests, confirming that high ME is fully correlated with the presence of a porous matrix, phyllosilicate fragments with totally and/or partially preserved foliation and non-sintered phases (amorphous and vitreous).

The unused “reference” samples U1, U3 and U5 show some differences, with sample U1 showing a greater amount of porous mass with less porosity, when compared to the detached samples (D1 to D5), and fewer vitreous mass cores (or lumps). These vitreous mass cores have more diffuse edges for this sample, which indicates a greater tendency towards homogeneity (Figure 11, Figure 12, Figure 13, Figure 14, Figure 15 and Figure 16).

The U3 sample is the most similar to the detached samples, as it contains very distinct vitreous mass cores immersed in a porous mass with greater porosity (when compared to the other unused samples), as well as having phases similar to phyllosilicates, as found in the detached samples, suggesting detachment potential. The EDS spot microanalysis (Figure 19, Figure 20 and Figure 21) shows the differences between the Al and Si peaks for the phyllosilicates (Figure 18a—red arrow) compared to the mass (Figure 18b—red arrow) and the surrounding porous mass (Figure 18b—green arrow), also indicative of high expansion potential [28].

Sample U5 (Figure 22) contains some very distinct vitreous cores, with well-marked edges, as observed in samples U3 and the other detached samples, but with a less porous surrounding mass when compared to the detached samples. No phyllosilicate-like phases were found in this sample.

By comparing the microstructure of the detached ceramic slabs with the unused ones, it can be seen that the samples of the detached tiles have a more porous mass and a greater number of vitreous mass cores with detached edges (heterogeneity). None of the samples showed the presence of mullite or any other crystalline structure.

All the setting mortars showed satisfactory results in terms of their hydration reactions, indicating that they were properly prepared and that the quantity of binders required for good adhesion to the back of the ceramic tile samples was adequate.

## 5. Conclusions

This study investigated samples of ceramic tiles and their adhesive mortars that showed detachment after laying with unused samples of the same conformation (dry-pressed tiles from group BIIb).

The standard test did not reach the suggested normative limit of 0.6 mm/m in any of the tests carried out. The tests therefore showed that either the normative test is unreliable for predicting future hygroscopic expansion that could lead to the collapse of ceramic tiles, or the suggested value of 0.6 mm/m should be reduced, based on the study of real cases by analyzing detached tiles and their expansion.

In the mineralogical analysis by X-ray diffraction and SEM, as well as in thermal analysis, a large number of amorphous phases, high porosity and the absence of mullite crystalline phases were observed in the detached pieces (D1 to D5) and in U2 for all the samples.

The XRD, TGA/DTA and XRF tests combined with the SEM tests were consistent with the results of the ME tests carried out on the detached ceramic tiles. The SEM tests applied to the autoclave ME tests are important for predicting future expansion of unused parts. It is recommended that further tests be conducted comparing the XRF tests with the autoclave ME tests of the ceramic slabs to confirm the results found in the literature where the alumina/silica and alkali oxides/alumina ratios are indicators of potential expansion.

## Figures and Tables

**Table 1:** Specifications of adhesive mortars type AC II according to the ABNT NBR 14,081 standard.

Adhesive mortars industrialized for the settlement of ceramic tiles type AC II (C1)	Industrialized adhesive mortar with adhesive characteristics that allow it to absorb the existing stresses in internal and external floor and wall coverings subject to cycles of thermo-hygrometric variation and wind action.
Bond strength at 28 days	
Initial tensile adhesion strength	≥0.5 MPa
Tensile adhesion strength after water immersion	≥0.5 MPa
Tensile adhesion strength after heat aging	≥0.5 MPa
Open time	≥20 min
Adhesion mechanism	Preferably mechanical/chemical

**Table 2:** Specifications of ceramic tiles type of BIIb according to the ABNT NBR ISO 13,006 standard.

Group BIIb	Manufacturing or forming method “B” (dry pressing) and water absorption ranging from 6% to 10%
Forming	Dry-pressed
Manufacturing method	Dry process
Glazed	(GL)
Use	For floor and wall coverings in internal areas
Dimensions	40 × 40 cm and 48 × 48 cm
National manufacture	Santa Gertrudes region (SP, Brazil)

**Table 3:** Identification of the tested samples and the tests carried out.

Sample	Situation	Mix Proportion	$E_{v}$/ME/CR *	DTA/TGA	XRD	XRF	SEM	EDS
DAM-1 to 5	Detached	X	---	X	X	---	---	---
DCT-1 to 5	Detached	---	X	X	X	X	X	---
UCT-1 to 5	Unused	---	X	---	---	---	X	X

Legend: * Ev: Water Absorption. ME: Moisture Expansion. CR: Crazing Resistance. X: Carried out. ---: Not carried out.

**Table 4:** Water absorption test results (%).

CT	Sample					Average
	1	2	3	4	5	
D	7.6	7.7	7.3	7.3	7.4	7.5
U	7.7	7.3	7.2	8.2	6.6	7.4

**Table 5:** Crazing resistance after autoclaving.

Sample (CT)	Crazing
D1–D5	YES
U1	NO
U2	YES
U3–U5	NO

**Table 6:** Mass losses from the TGA/DTA curves in the detached ceramic tiles (DCT) samples.

Sample CT	Mass Loss as a Function of Temperature Range						Total Loss (%)
	°C	23–150	150–435	435–850	850–1000	1000–1100	
D1	%	0.28	0.46		0.02	0.10	0.86
D2	%	0.58	0.65		0.03	0.04	1.30
D3	%	0.33	0.67		0.02	0.05	1.07
D4	%	0.30	0.63		0.03	0.03	0.99
D5	%	0.54	0.72		0.03	0.06	1.35

**Table 7:** Interpretation of mass loss as a function of temperature range for detached ceramic tiles (DCT).

25–150 °C	Water-free
150–850 °C	Absorbed water
	Dehydroxylation of goethite and residual clay minerals
850–1100 °C	Decomposition of phyllosilicates
	Formation of intermediate phase
	Formation of mullite and cristobalite
>1100 °C	Samples melted

**Table 8:** X-ray fluorescence chemical analysis results.

Determination	Results (%)				
	D1	D2	D3	D4	D5
Fire Loss (FL)	0.7	1.1	1.1	1.0	1.3
SiO_2_	73.6	72.6	72.5	72.1	71.5
Al_2_O_3_	12.6	13.4	11.9	12.3	12.4
Fe_2_O_3_	5.1	5.2	5.9	5.1	6.7
K_2_O	3.2	3.1	3.6	3.2	3.1
Na_2_O	1.4	1.3	1.5	1.4	1.3
MgO	1.2	1.3	1.2	1.2	1.2
CaO	0.9	0.9	1.0	0.9	1.1
TiO_2_	0.6	0.6	0.7	0.6	0.8
ZnO	0.2	0.1	0.2	0.1	0.2
BaO	0.1	0.1	0.1	0.1	0.1
MnO	0.1	0.1	0.1	0.1	0.1
ZrO_2_	<0.1	<0.1	<0.1	<0.1	<0.1
Cr_2_O_3_					
P_2_O_2_					
CuO					
SrO					

**Table 9:** Mass losses from TGA/DTA curves on detached adhesive mortar (DAM) samples.

DAM Sample	Mass Loss as a Function of Temperature Range									Total Loss (%)
	°C	23–60	60–200	200–260	260–320	320–400	400–470	470–635	635–1000	
AM1	%	0.76	2.49	0.39	0.26	0.37	0.28	1.02	1.27	6.84
AM2	%	0.60	1.64	0.3	0.27	0.45	2.28		4.44	9.98
AM3	%	0.37	2.58	0.38	0.24	0.43	1.72		1.55	7.27
AM4	%	1.03	3.28	0.43	0.42	0.59	0.43	2.08	4.15	12.40
AM5	%	0.84	2.88	0.24	0.28	0.41	0.44	1.40	2.33	8.82

**Table 10:** Interpretation of the mass losses according to the temperature range for the DAM.

ΔT (°C)	Interpretation of Mass Loss as a Function of Temperature Range
23–60	Loss of free water and adsorption water
60–~220	Dehydration of sulfate compounds (gypsum, ettringite (AFt), calcium monosulfoaluminate (AFm) and others) and C-S-H
~220–~260	Additional dihydroxylation of calcium monosulfoaluminate (AFm)
~260–~310	Release and/or loss of water from gels and dihydroxylation of aluminates
~310–400	Dehydration of Mg(OH)_2_ (brucite)
400–470	Dihydroxylation of Ca(OH)_2_ (portlandite)
470–635	Dehydration and/or additional dihydroxylation of C-S-H and decomposition of poorly crystallized carboaluminates and carbonates
635–1000	Decomposition of calcium carbonate

**Table 11:** Results of XRD analysis of DAM.

DAM Sample	Mineralogical Compounds or Phases
AM1	Quartz, feldspar (plagioclase and alkali), mica, calcite, portlandite, gypsum, brownmillerite, ettringite (AFt), anhydrous silicates (C_3_S and C_2_S) and dolomite.
AM2	Quartz, feldspar (plagioclase and alkali), brownmillerite, calcite, dolomite, ettringite (AFt) and portlandite [Ca(OH)_2_)].
AM3	Quartz, feldspar (plagioclase and alkali), mica, anhydrous silicate (C_3_S), ettringite (AFt), calcite and gypsum.
AM4	Quartz, feldspar (plagioclase and alkali), brownmillerite, anhydrous silicates (C_3_S and C_2_S), portlandite, dolomite, calcite, ettringite (AFt) and gypsum.
AM5	Quartz, feldspar (plagioclase and alkali), calcite, dolomite, anhydrous silicate (C_3_S), ettringite (AFt) and mica.

**Table 12:** Chemical analysis results for DAM (%).

Determination	AM1		AM2		AM3		AM4		AM5	
	Original	Non- Volatile	Original	Non- Volatile	Original	Non- Volatile	Original	Non- Volatile	Original	Non- Volatile
Humidity	2.94	----	1.78	----	3.20	----	3.95	----	4.71	----
Fire loss	4.43	----	8.20	----	5.21	----	9.88	----	6.39	----
Insoluble residue	78.30	84.50	73.20	81.30	76.80	83.90	62.20	72.20	73.00	82.10
Silicic anhydride (SiO_2_)	3.13	3.38	2.62	2.91	3.43	3.74	3.64	4.22	3.77	4.24
Iron and aluminum oxides (R2O3)	2.19	2.36	1.81	2.01	2.28	2.49	2.75	3.19	2.54	2.86
Calcium oxide (CaO)	7.76	8.38	10.20	11.30	7.93	8.66	14.20	16.50	8.29	9.33
Magnesium oxide (MgO)	0.92	0.99	1.65	1.83	0.95	1.04	2.12	2.46	1.31	1.47
Sulfuric anhydride (SO_3_)	0.38	0.41	0.46	0.51	0.40	0.44	0.64	0.74	0.36	0.40
Carbon dioxide (CO_2_)	1.78	----	6.45	----	2.58	----	5.91	----	3.40	----
Sulfide (S^2−^)	<0.01	----	<0.01	----	<0.01	----	<0.01	----	<0.01	----

**Table 13:** Proportion mix reconstruction results.

DAM Sample	Calculated Parameters	Cement	Aggregate Siliceous	Binder: Aggregate
AM1	Constituents (%)	16.3	83.7	1:5.1
	Prop. in mass	1	5.1	
AM2	Constituents (%)	17.3	82.7	1:4.4
	Prop. in mass	1	4.8	
AM3	Constituents (%)	17.9	82.1	1:4.6
	Prop. in mass	1	4.6	
AM4	Constituents (%)	25.4	74.6	1:2.6
	Ratio in mass	1	2.9	
AM5	Constituents (%)	20.3	79.7	1:3.9
	Ratio in mass	1	3.9	

**Table 14:** Constituents of the Portland cements and lime adopted as reference.

Reference	Original Base (%)					Non-Volatile Base (%)			
	PF	SiO_2_-RI	Ca O	Mg O	SO_3_	SiO_2_-RI	Ca O	Mg O	SO_3_
CP II F	5.8	17.2	60.8	3.33	2.66	18.2	64.6	3.54	2.82
CP II E	3.83	19.8	52.7	5.37	1.42	21.3	54.8	5.58	1.48

**Table 15:** Calculated parameters SO_3_ of five mortar types as a function of cement content.

Ratio by Volume	Cement Content in Mortars (%)	SO_3_ Limit Calculated per Mortar (%)
1:3	25.8	1.03
1:4	20.7	0.83
1:5	17.3	0.69
1:6	14.8	0.59
1:8	11.5	0.46

## Data Availability

The original contributions presented in the study are included in the article/supplementary material, further inquiries can be directed to the corresponding author.

## Supplementary Materials

The following supporting information can be downloaded at: https://www.mdpi.com/article/10.3390/ma18020465/s1, File S1: TGA/DTA of detached ceramic tiles (DCTs); File S2: XRD of detached ceramic plates (DCTs); File S3: TGA/DTA of detached adhesive mortars (DAMs); File S4: XRD of detached adhesive mortars (DAMs); File S5: EDS of unused ceramic tile (UCT).
