# Supplementary material for: Investigating the Detachment of Glazed Ceramic Tiles Used in Buildings: A Brazilian Case Study
Source: Materials (Basel). 2025 Jan 20;18(2):465. doi: 10.3390/ma18020465 (PMC11766741; doi:10.3390/ma18020465)
Supplement: Supplementary file 1 [file materials-18-00465-s001.zip › Supplementary File S1.pdf]

SUPPLEMENTARY FILE S1 – TGA/DTA OF DETACHED CERAMIC TILES (DCT)

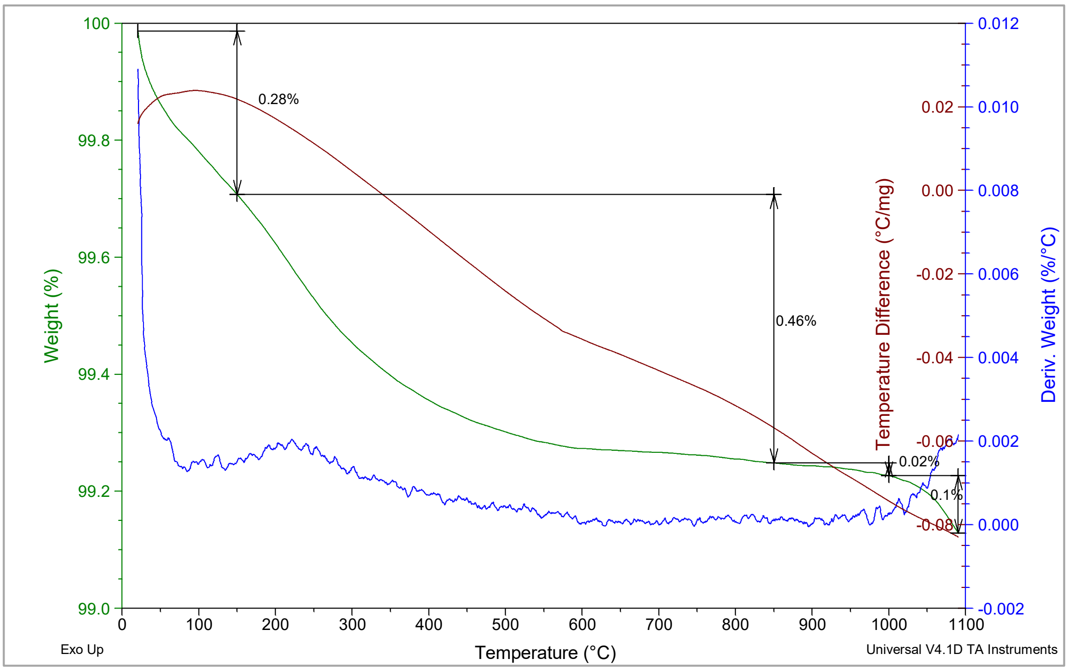

Figure A1. TGA/DTA curves of sample D1. Between 25° and 150 °C, free water loss of 0.28%. Between 150° and 850 °C, loss of adsorbed water with dehydroxylation of goethite and residual clay minerals of 0.46%. Between 400° and 800 °C, it denotes a discrete transformation of the phyllosilicate present, with formation of an intermediate phase. Between 850° and 1100 °C, the exothermic peaks are related to the nucleation of mullite and cristobalite. Melting above 1100 °C. Total mass loss of 0.86%.

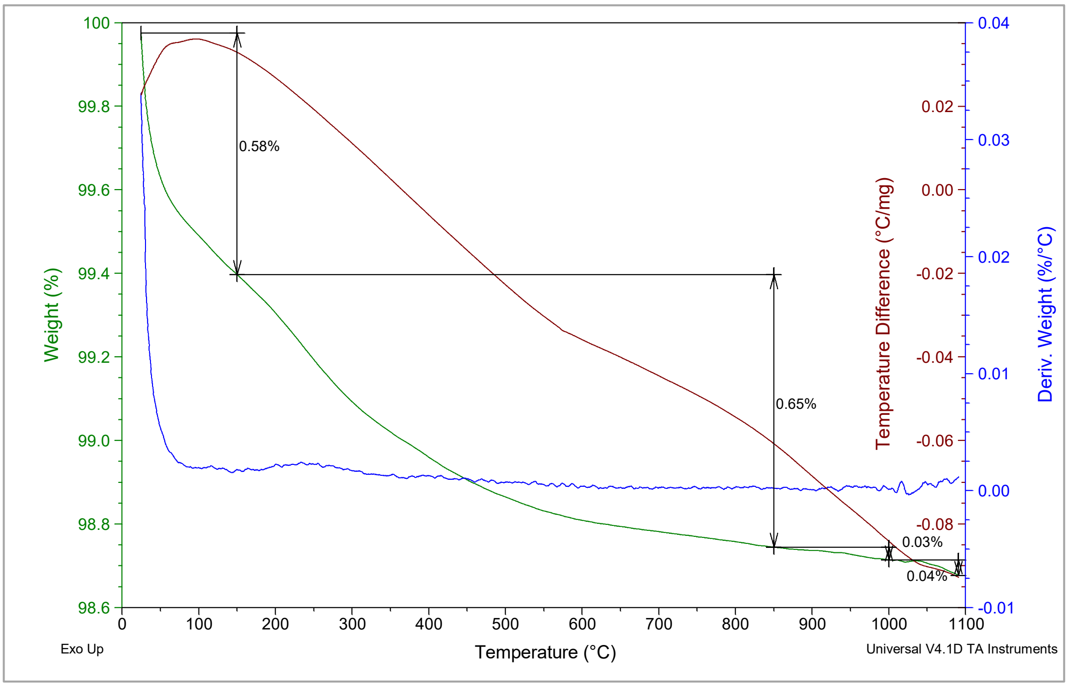

Figure A2. TGA /DTA curves of sample D2. Between 25° and 150 °C, free water loss of 0.58%. Between 150° and 850 °C, loss of adsorbed water with dehydroxylation of goethite and residual clay minerals of 0.65%. Between 400° and 800 °C, it denotes a discrete transformation of the phyllosilicate present, with formation of an intermediate phase. Between 850° and 1100 °C, the exothermic peaks are related to the nucleation of mullite and cristobalite. The sample presented melting above 1100 °C. Total mass loss of 1.30%.

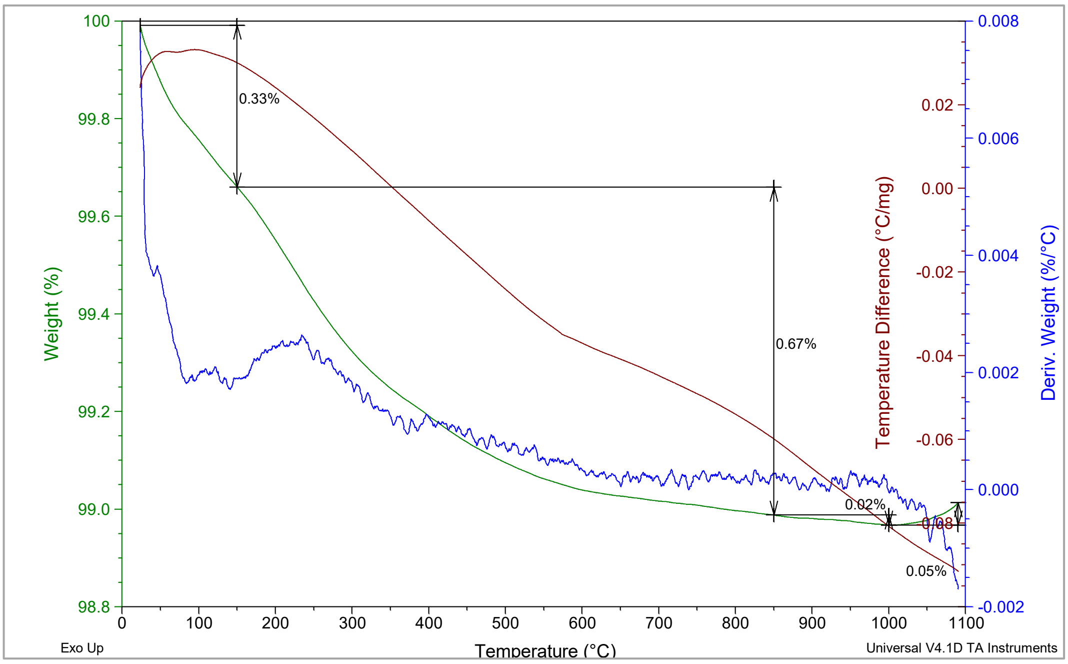

Figure A3. TGA /DTA curves of sample D3. Between 25° and 150 °C, free water loss of 0.33%. Between 150° and 850 °C, loss of adsorbed water with dehydroxylation of goethite and residual clay minerals of 0.67%. Between 400° and 800 °C, it denotes a discrete transformation of the phyllosilicate present, with formation of an intermediate phase. Between 850° and 1100 °C, the exothermic peaks are related to the nucleation of mullite and cristobalite. The sample presented melting above 1100 °C. Total mass loss of 1.07%.

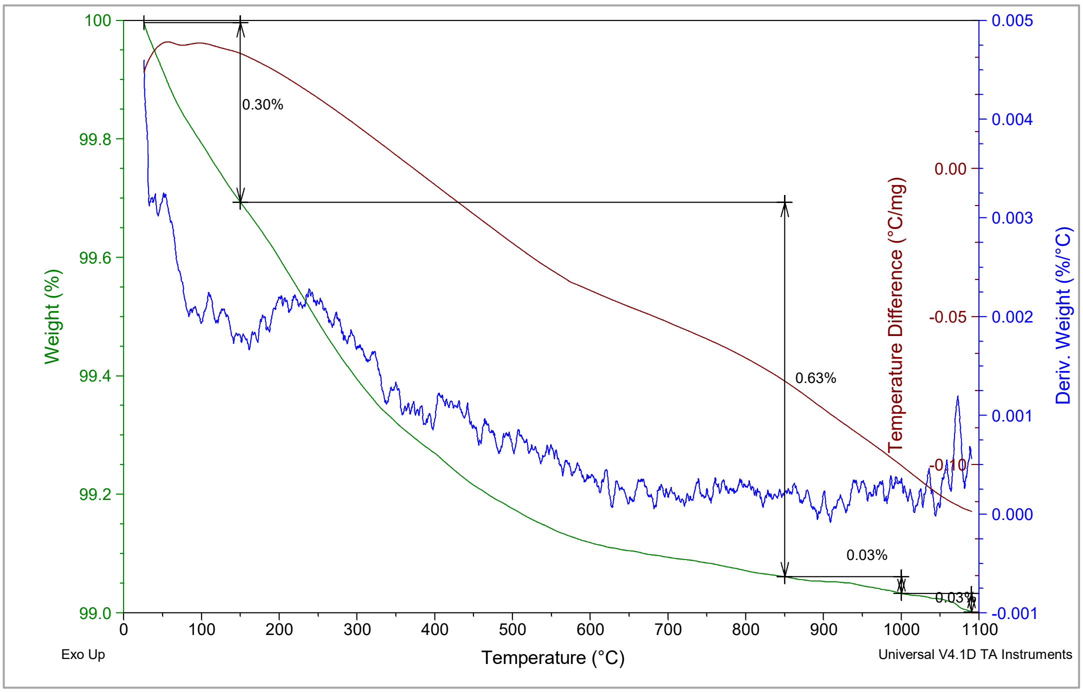

Figure A4. TGA /DTA curves of sample D4. Between 25° and 150 °C, free water loss of 0.30%. Between 150° and 850 °C, loss of adsorbed water with dehydroxylation of goethite and residual clay minerals of 0.63%. A slight endothermic reaction is observed between 400° and 800 °C, which denotes possible transformation of either residual kaolinite (more pronounced and visible in the analysis of sample D4) or another phyllosilicate present, with formation of an intermediate phase. Between 850° and 1100 °C, the exothermic peaks are related to the nucleation of mullite and cristobalite. The sample presented melting above 1100 °C. Total mass loss of 0.99%.

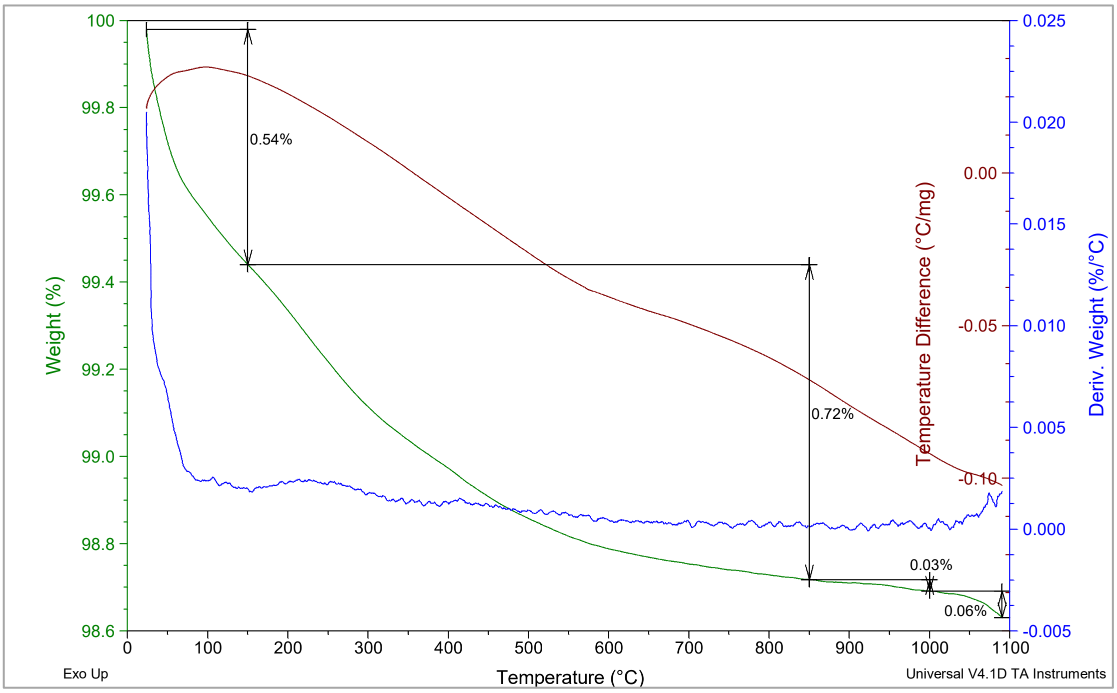

Figure A5. TGA /DTA curves of sample D5. Between 25° and 150 °C, free water loss of 0.54%. Between 150° and 850 °C, loss of adsorbed water with dehydroxylation of goethite and residual clay minerals of 0.72%. Between 400° and 800 °C, it denotes a discrete transformation of the phyllosilicate present, with formation of an intermediate phase. Between 850° and 1100 °C, the exothermic peaks are related to the nucleation of mullite and cristobalite. The sample presented melting above 1100 °C. Total mass loss of 1.35%.
