# Supplementary material for: Investigating the Detachment of Glazed Ceramic Tiles Used in Buildings: A Brazilian Case Study
Source: Materials (Basel). 2025 Jan 20;18(2):465. doi: 10.3390/ma18020465 (PMC11766741; doi:10.3390/ma18020465)
Supplement: Supplementary file 1 [file materials-18-00465-s001.zip › Supplementary File S2.pdf]

SUPPLEMENTARY FILE S2 – XRD OF DETACHED CERAMIC PLATES (DCT)

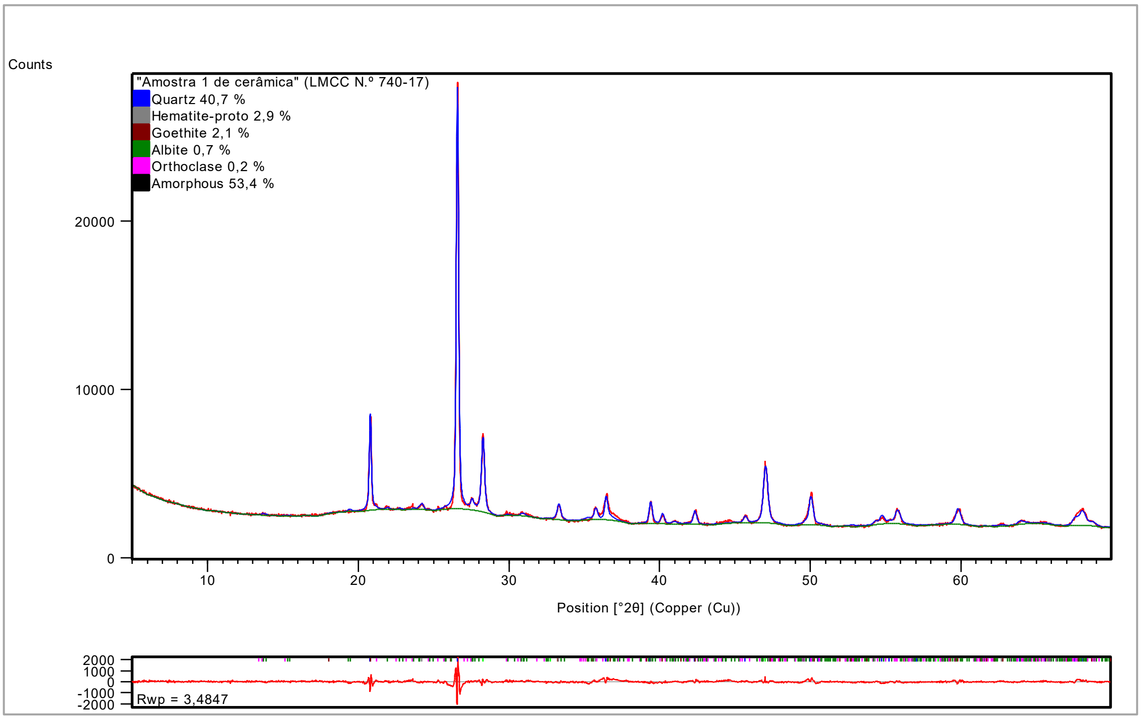

Figure B1. X-ray diffractogram of sample D1

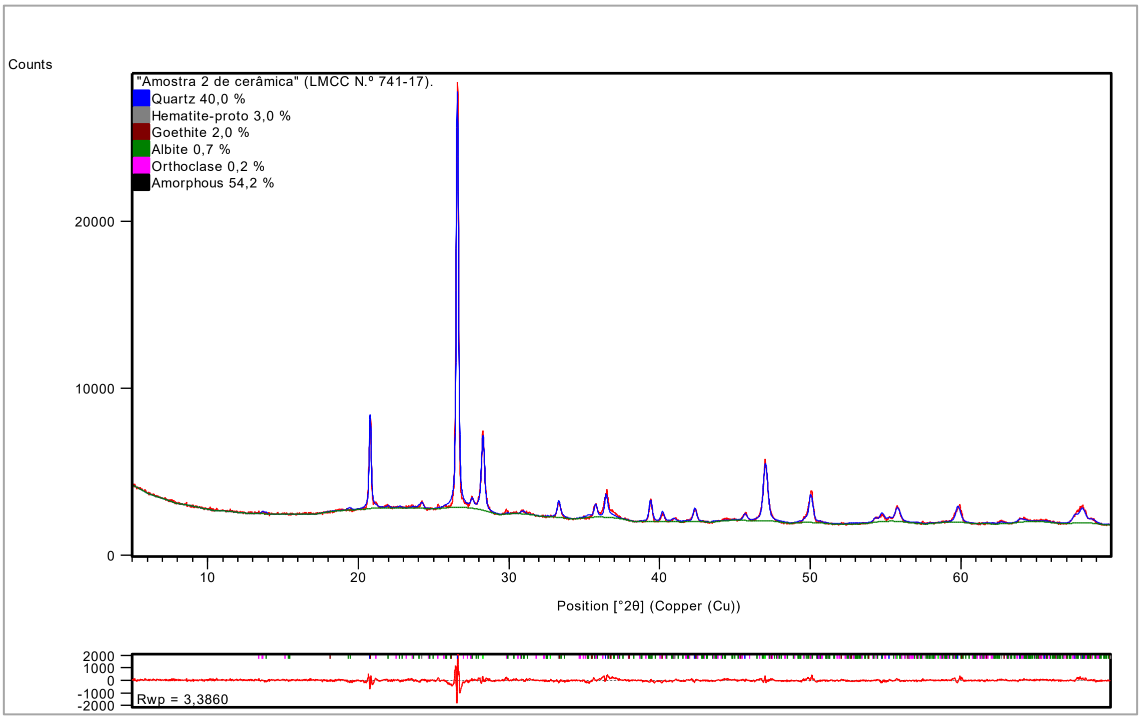

Figure B2. X-ray diffractogram of sample D2.

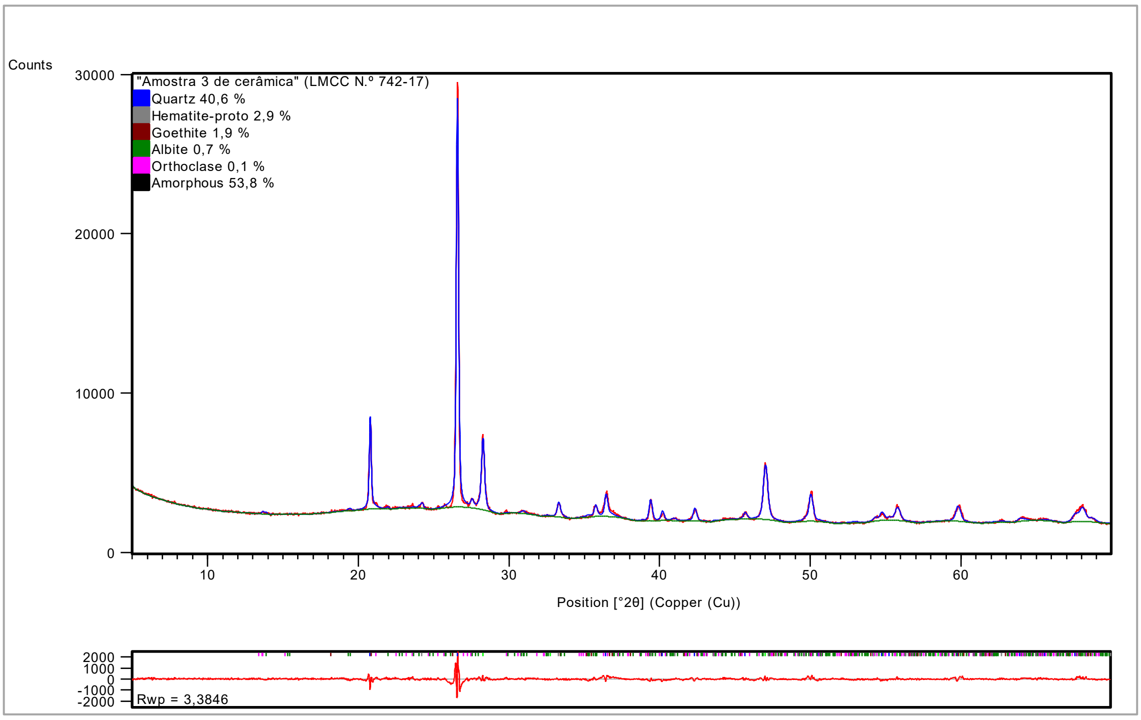

Figure B3. X-ray diffractogram of sample D3.

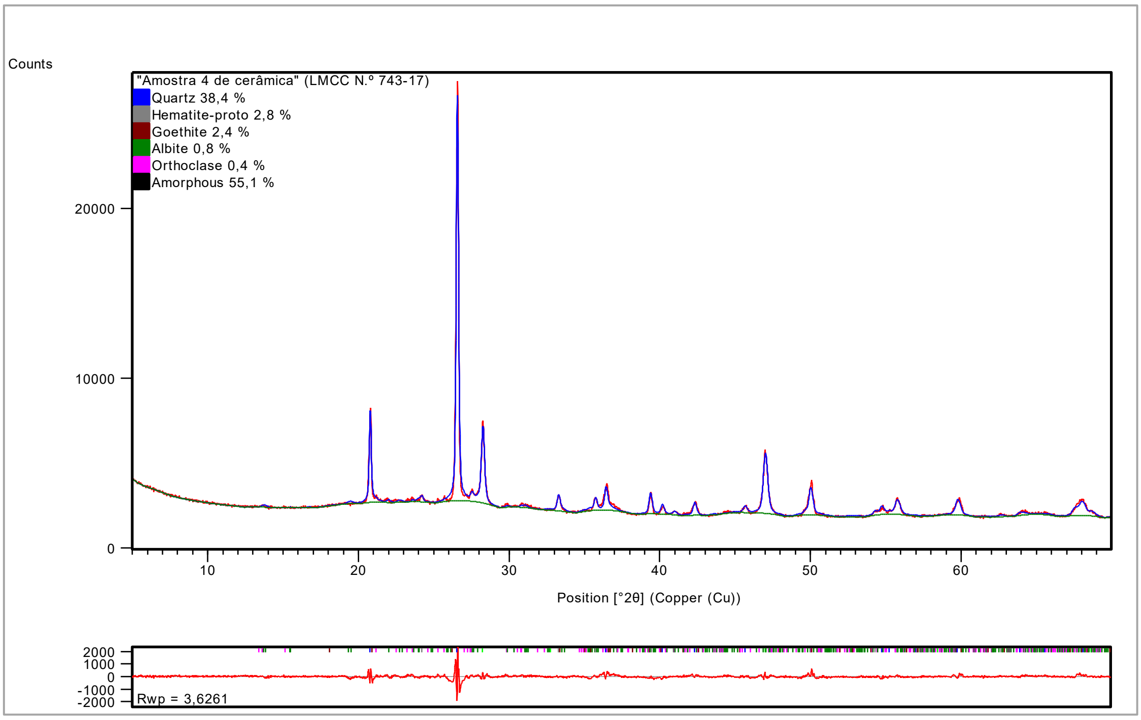

Figure B4. X-ray diffractogram of sample D4.

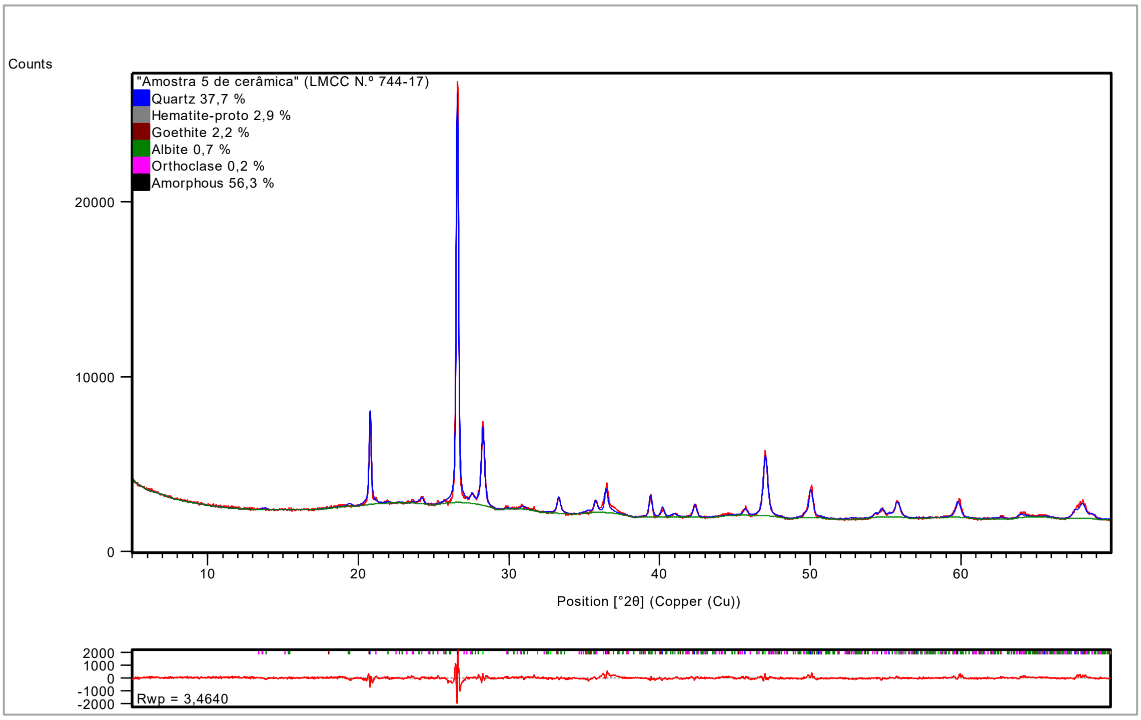

Figure B5. X-ray diffractogram of sample D5
