# Supplementary material for: Investigating the Detachment of Glazed Ceramic Tiles Used in Buildings: A Brazilian Case Study
Source: Materials (Basel). 2025 Jan 20;18(2):465. doi: 10.3390/ma18020465 (PMC11766741; doi:10.3390/ma18020465)
Supplement: Supplementary file 1 [file materials-18-00465-s001.zip › Supplementary File S3.pdf]

SUPPLEMENTARY FILE S3 – TGA/DTA OF DETACHED ADHESIVE MORTARS (DAM)

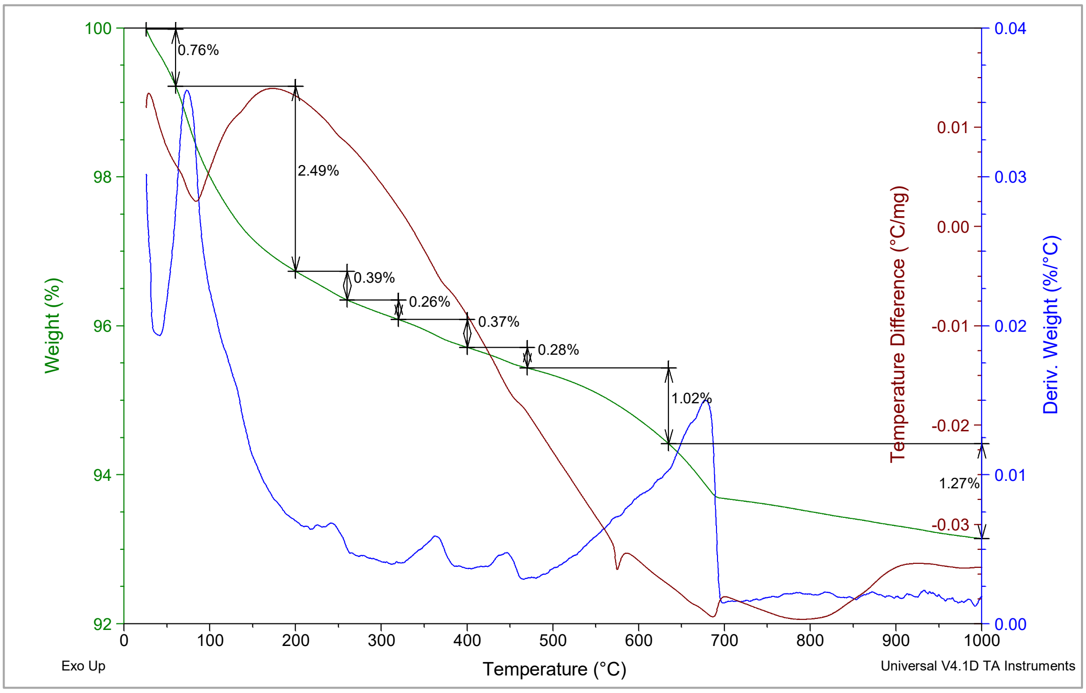

Figure C 1. TGA/DTA curves of sample AM1. Total mass loss of 6.84%.

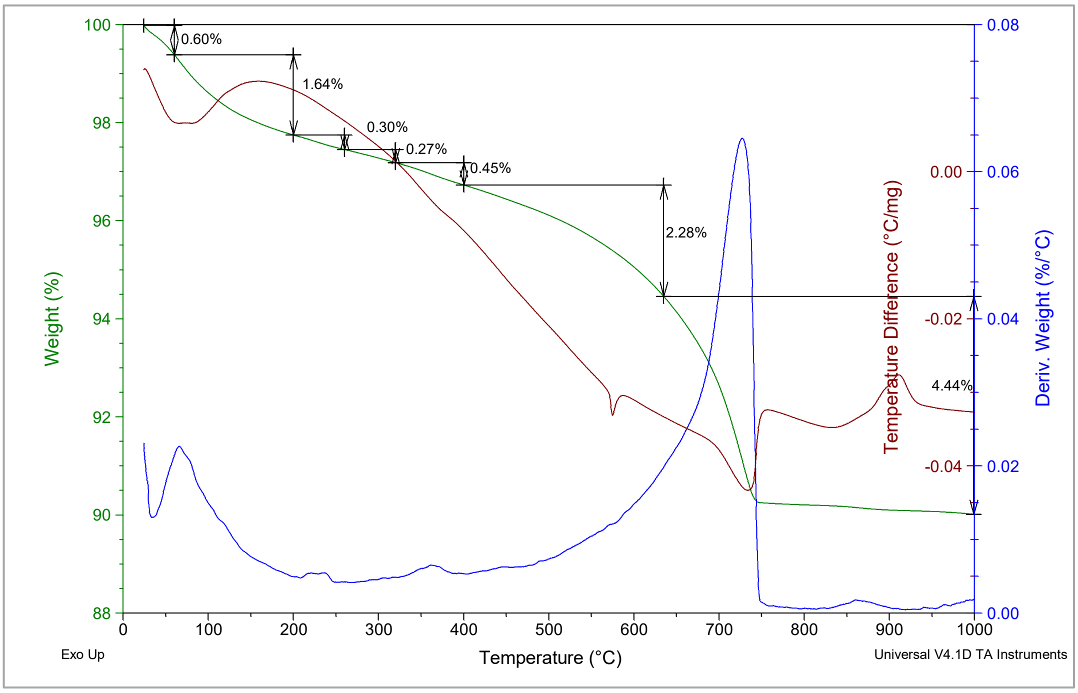

Figure C 2. TGA/DTA curves of sample AM2. Total mass loss of 9.98%.

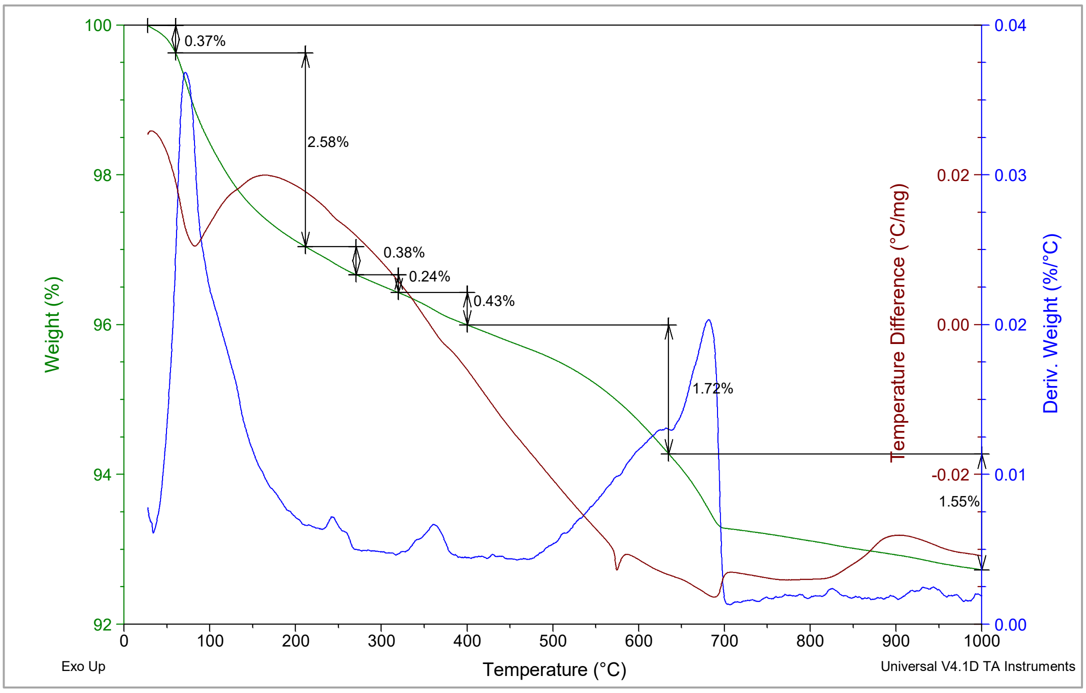

Figure C 3. TGA/DTA curves of sample AM3. Total mass loss of 7.27%.

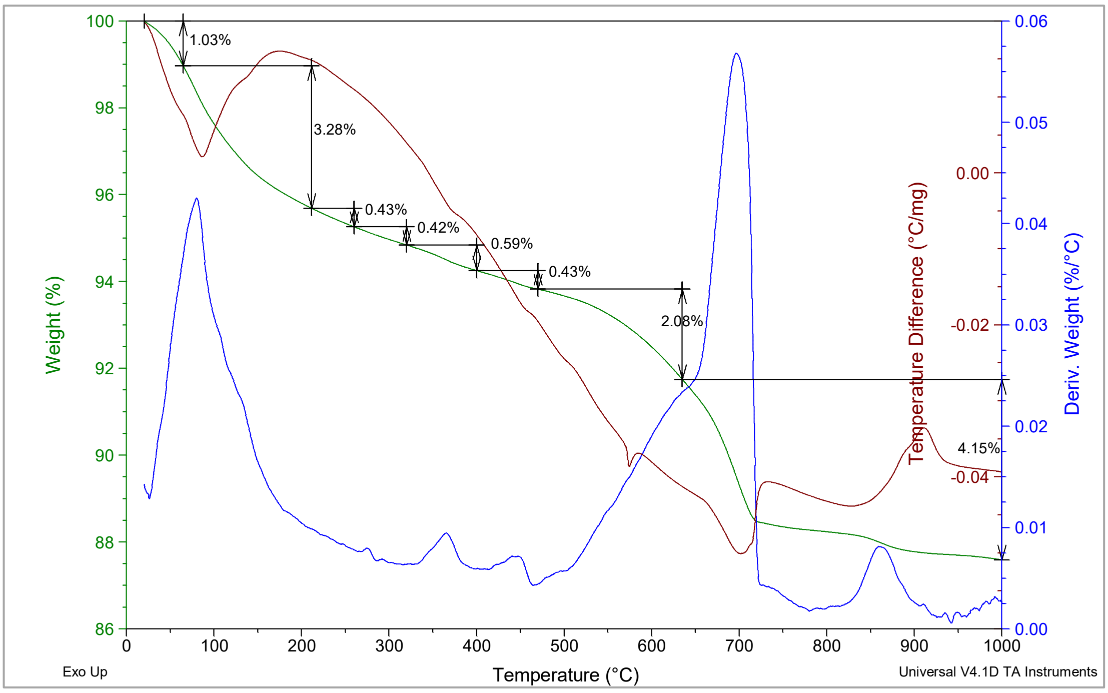

Figure C 4. TGA/DTA curves of sample AM4. Total mass loss of 12.4%.

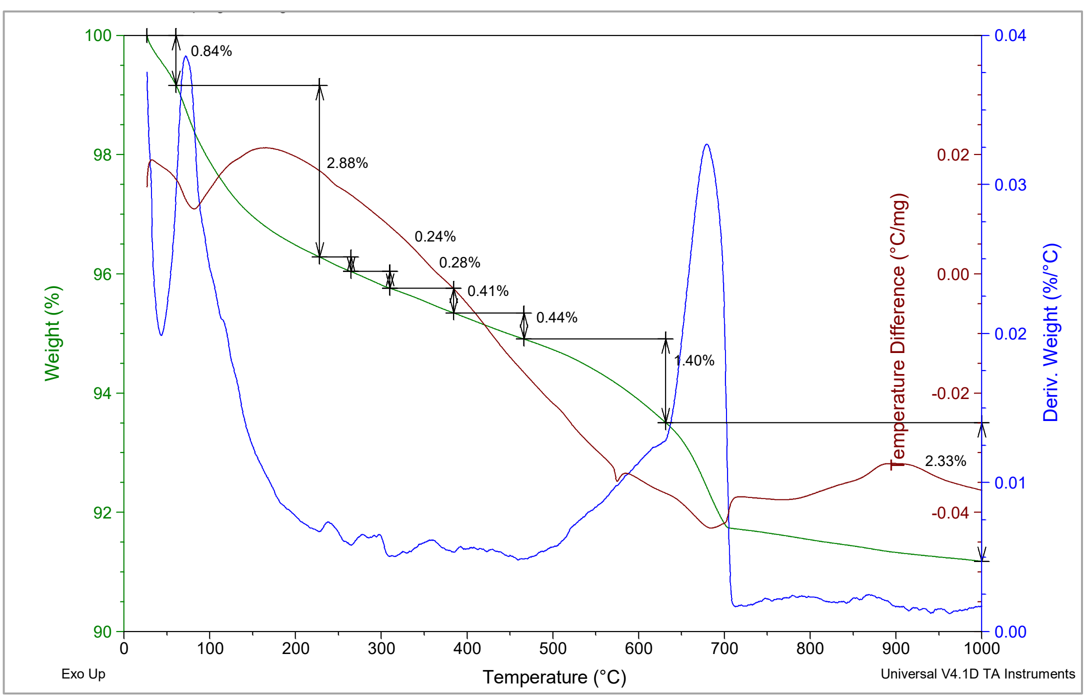

Figure C 5. TGA/DTA curves of sample AM5. Total mass loss of 8.82%.
