# Supplementary material for: Investigating the Detachment of Glazed Ceramic Tiles Used in Buildings: A Brazilian Case Study
Source: Materials (Basel). 2025 Jan 20;18(2):465. doi: 10.3390/ma18020465 (PMC11766741; doi:10.3390/ma18020465)
Supplement: Supplementary file 1 [file materials-18-00465-s001.zip › Supplementary File S4.pdf]

SUPPLEMENTARY FILE S4 – XRD OF DETACHED ADHESIVE MORTARS (DAM)

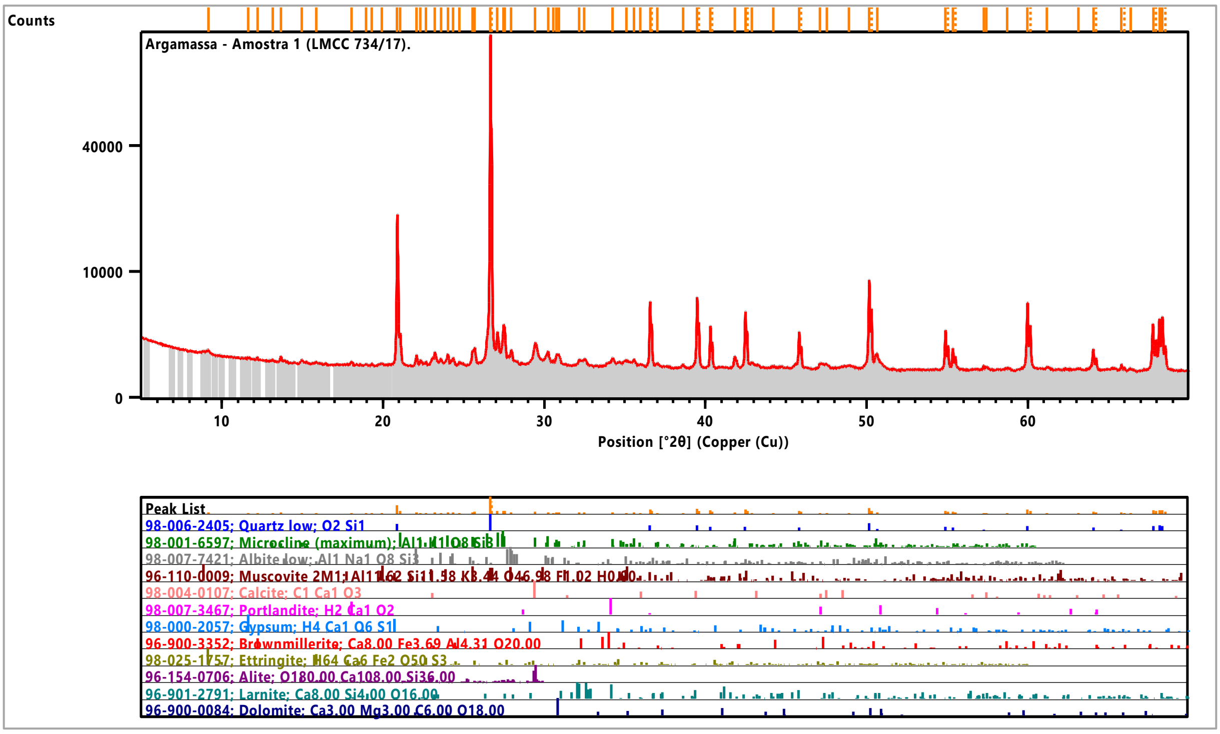

Figure D1. X-ray diffractogram of sample AM1

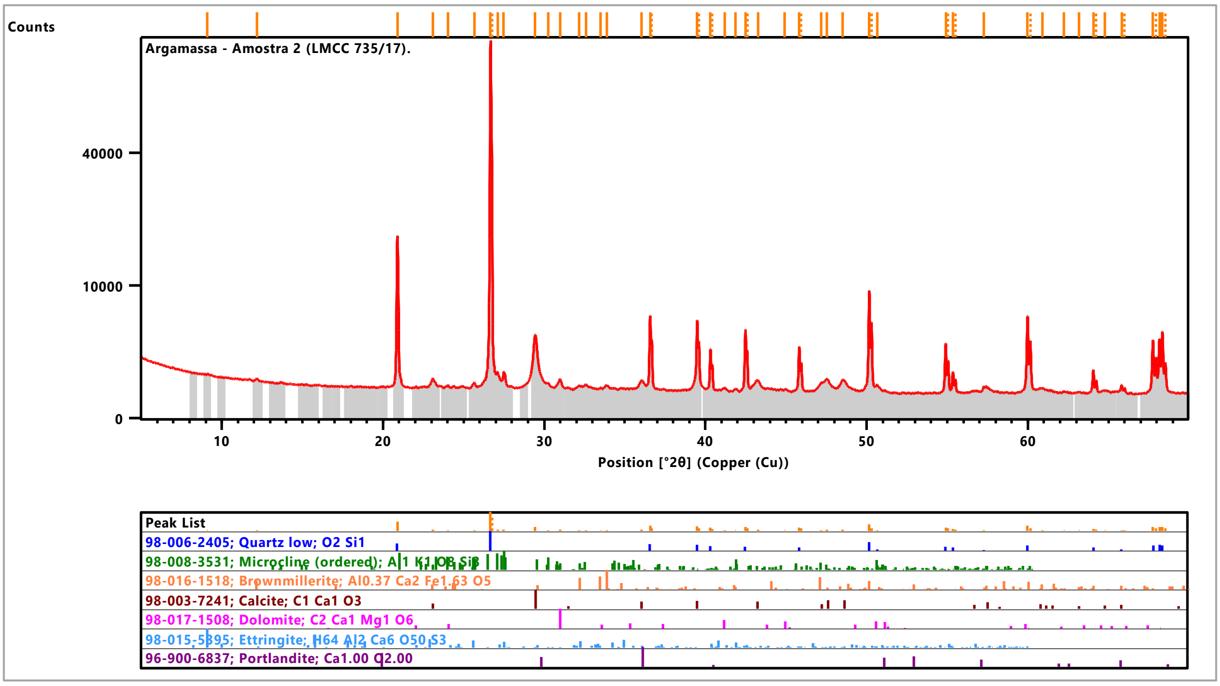

Figure D2. X-ray diffractogram of sample AM2

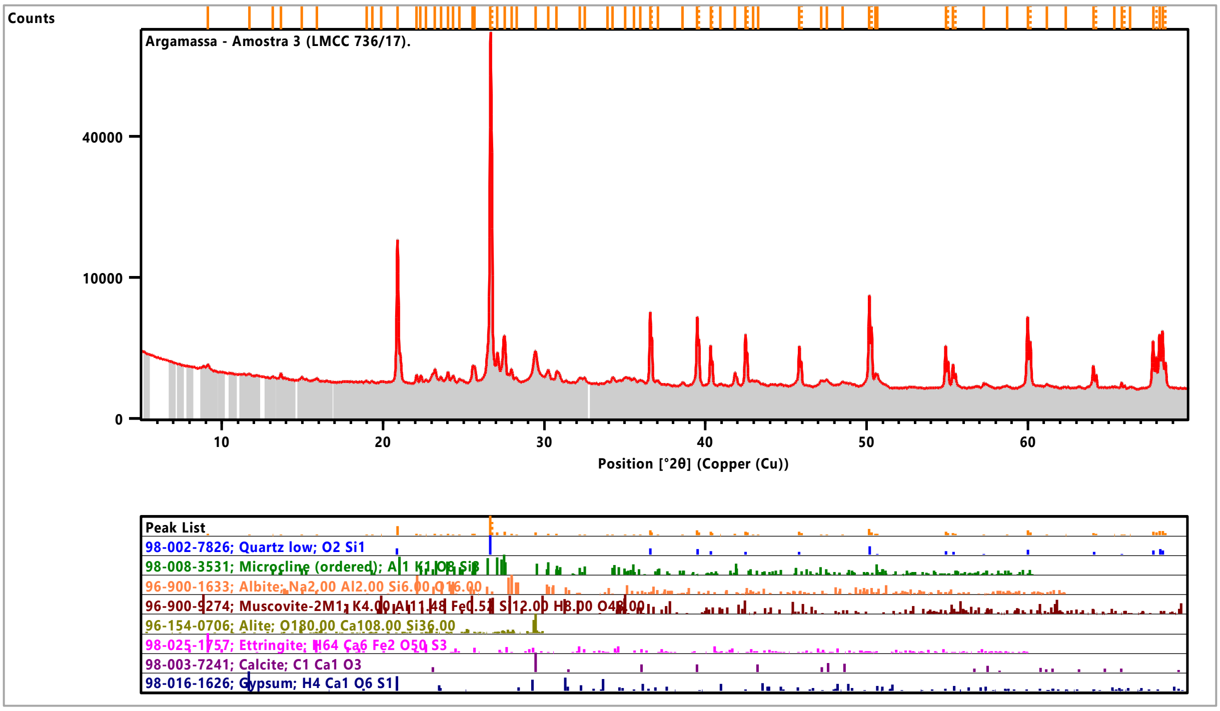

Figure D3. X-ray diffractogram of sample AM3

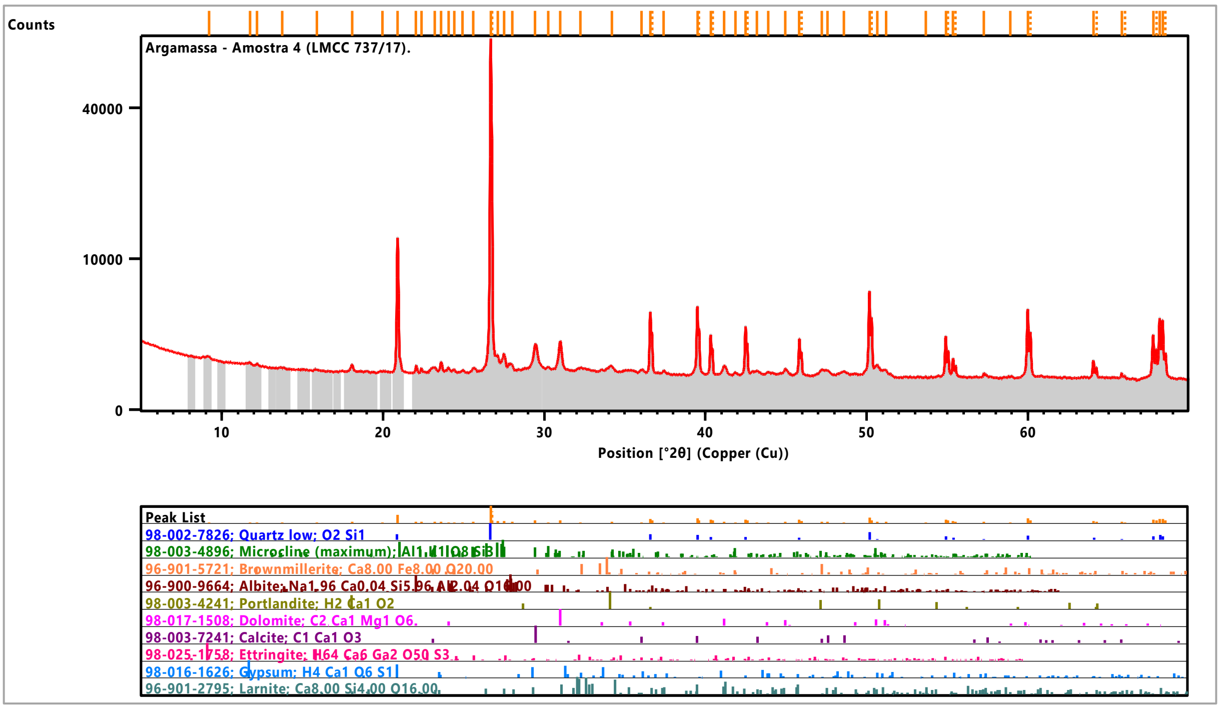

Figure D4. X-ray diffractogram of sample AM4

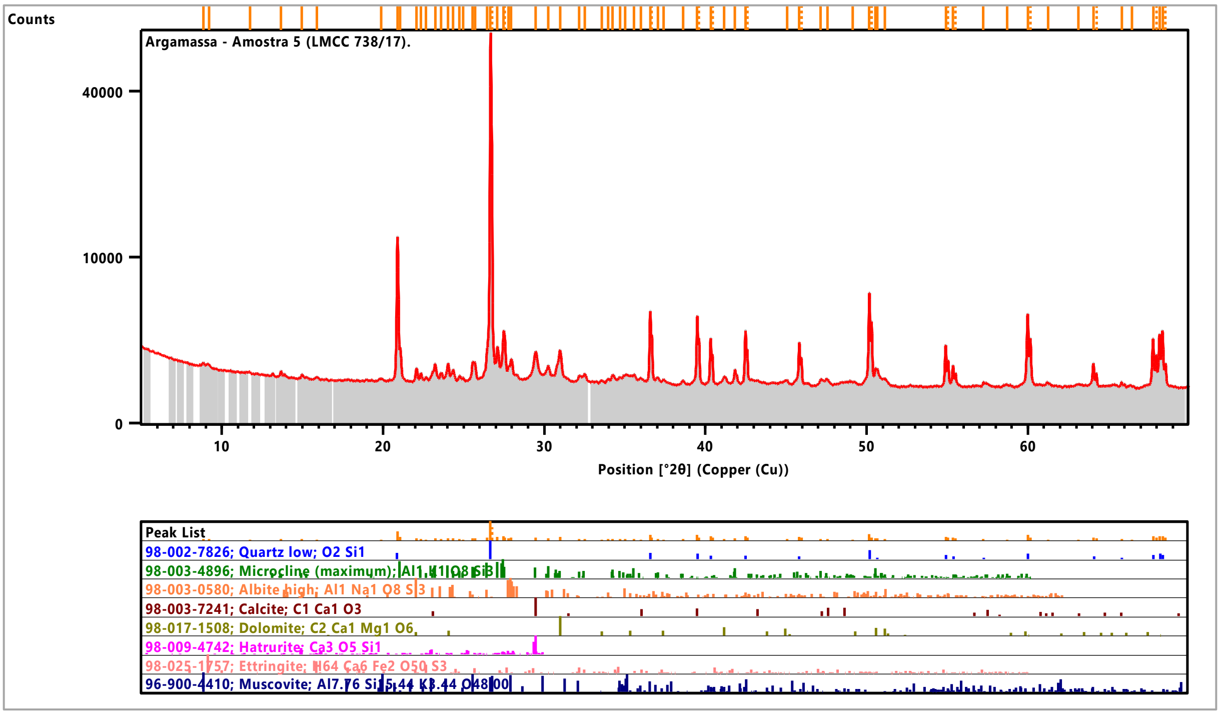

Figure D5. X-ray diffractogram of sample AM5
