# Supplementary material for: Investigating the Detachment of Glazed Ceramic Tiles Used in Buildings: A Brazilian Case Study
Source: Materials (Basel). 2025 Jan 20;18(2):465. doi: 10.3390/ma18020465 (PMC11766741; doi:10.3390/ma18020465)
Supplement: Supplementary file 1 [file materials-18-00465-s001.zip › Supplementary File S5.pdf]

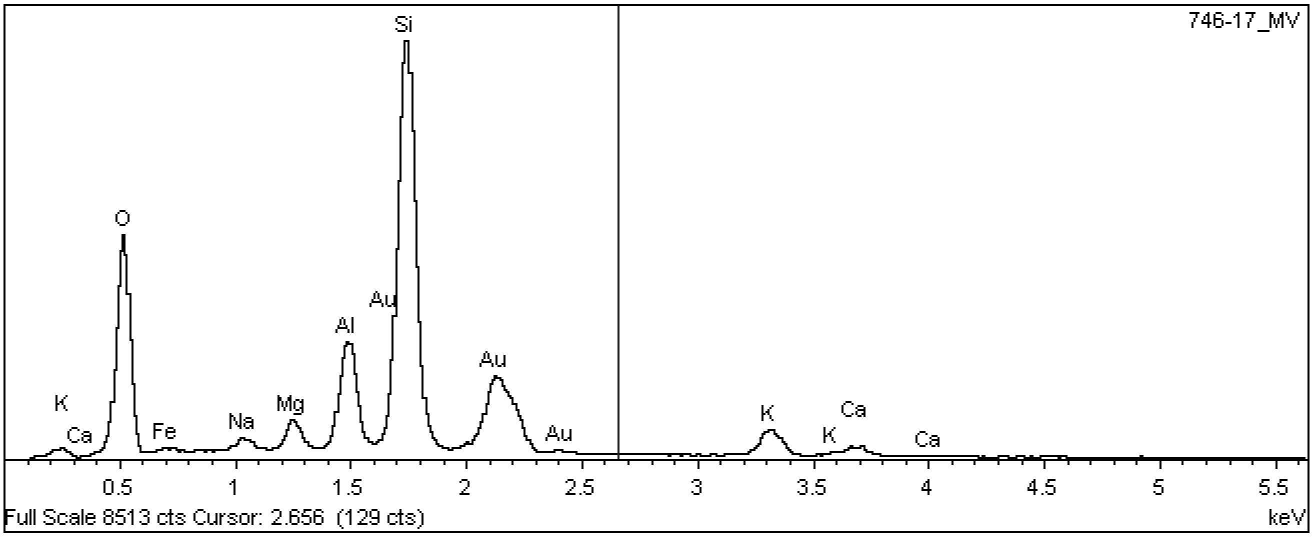

Spectrum processing :
Peak possibly omitted : 4.531 keV

Processing option : All elements analyzed (Normalised)
Number of iterations = 3

Standard :
O SiO2 1-Jun-1999 12:00 AM
Na Albite 1-Jun-1999 12:00 AM
Mg MgO 1-Jun-1999 12:00 AM
Al Al2O3 1-Jun-1999 12:00 AM
Si SiO2 1-Jun-1999 12:00 AM
K MAD-10 Feldspar 1-Jun-1999 12:00 AM
Ca Wollastonite 1-Jun-1999 12:00 AM
Fe Fe 1-Jun-1999 12:00 AM

Quantitative results

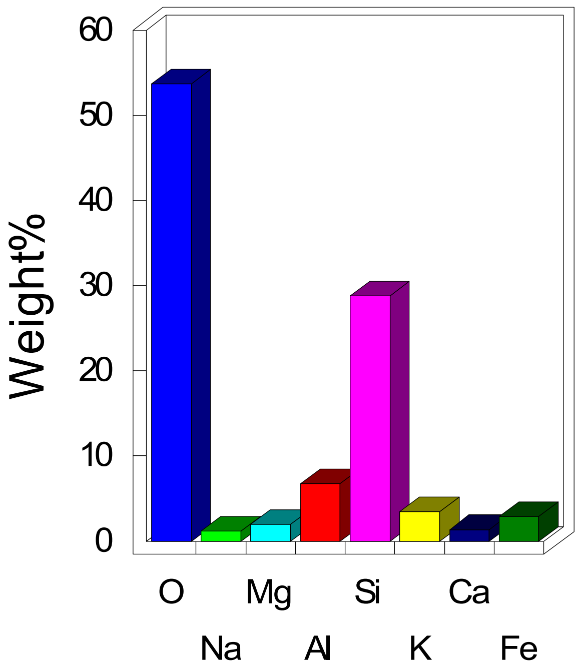

Element	Weight%	Atomic%
O K	53.68	68.00
Na K	1.15	1.02
Mg K	1.96	1.63
Al K	6.73	5.06
Si K	28.78	20.77
K K	3.45	1.79
Ca K	1.36	0.69
Fe K	2.88	1.05
Totals	100.00	

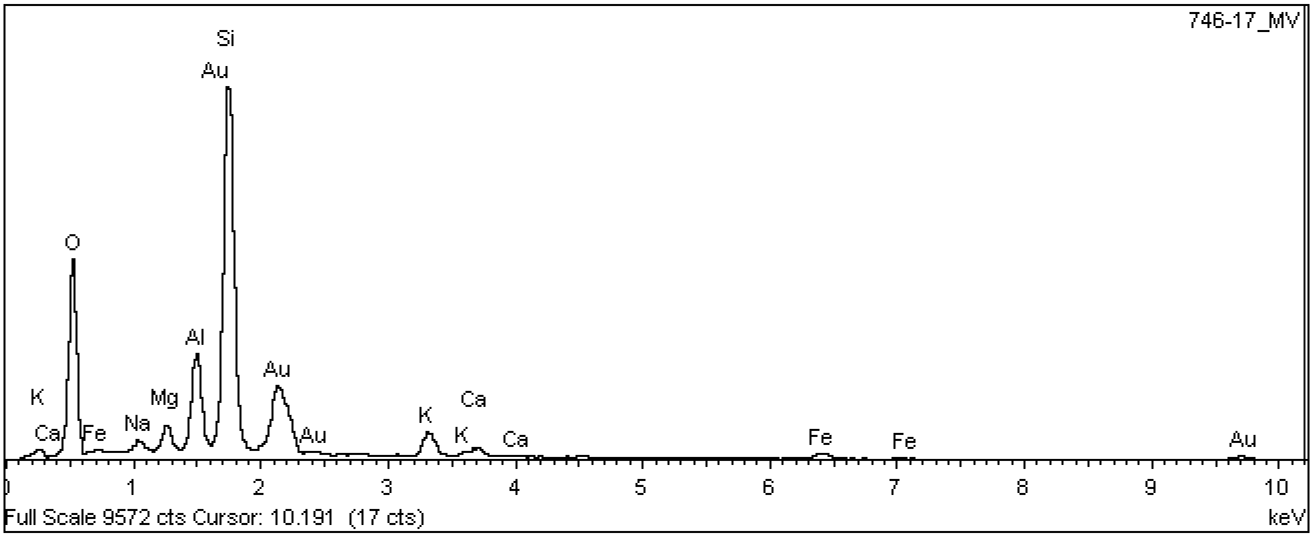

Spectrum processing :
Peak possibly omitted : 4.531 keV

Processing option : All elements analyzed (Normalised)
Number of iterations = 3

Standard :
O SiO2 1-Jun-1999 12:00 AM
Na Albite 1-Jun-1999 12:00 AM
Mg MgO 1-Jun-1999 12:00 AM
Al Al2O3 1-Jun-1999 12:00 AM
Si SiO2 1-Jun-1999 12:00 AM
K MAD-10 Feldspar 1-Jun-1999 12:00 AM
Ca Wollastonite 1-Jun-1999 12:00 AM
Fe Fe 1-Jun-1999 12:00 AM

Quantitative results

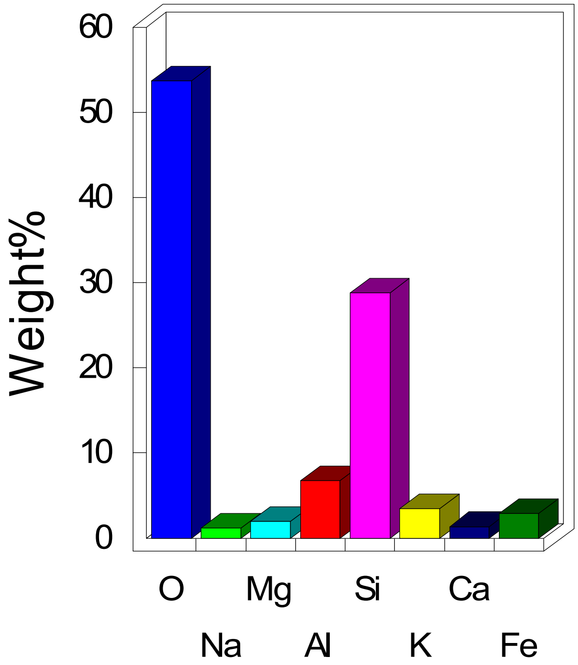

Element	Weight%	Atomic%
O K	53.68	68.00
Na K	1.15	1.02
Mg K	1.96	1.63
Al K	6.73	5.06
Si K	28.78	20.77
K K	3.45	1.79
Ca K	1.36	0.69
Fe K	2.88	1.05
Totals	100.00	

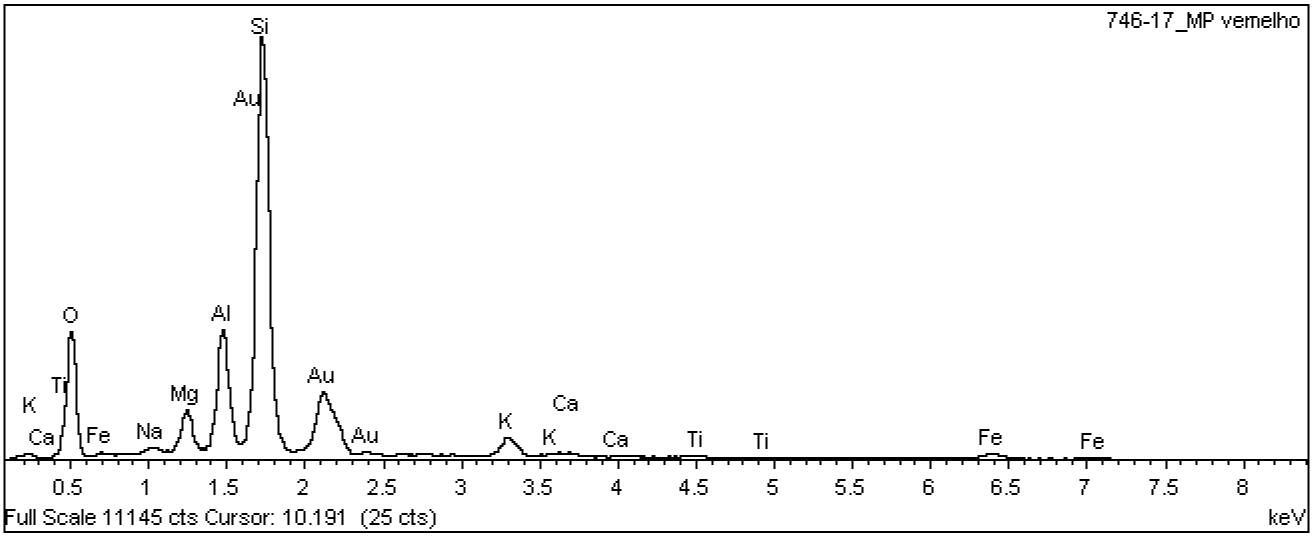

Spectrum processing :
No peaks omitted

Processing option : All elements analyzed (Normalised)
Number of iterations = 3

Standard :
O SiO2 1-Jun-1999 12:00 AM
Na Albite 1-Jun-1999 12:00 AM
Mg MgO 1-Jun-1999 12:00 AM
Al Al2O3 1-Jun-1999 12:00 AM
Si SiO2 1-Jun-1999 12:00 AM
K MAD-10 Feldspar 1-Jun-1999 12:00 AM
Ca Wollastonite 1-Jun-1999 12:00 AM
Ti Ti 1-Jun-1999 12:00 AM
Fe Fe 1-Jun-1999 12:00 AM

Element	Weight%	Atomic%
O K	43.73	58.47
Na K	0.59	0.55
Mg K	3.35	2.94
Al K	9.10	7.21
Si K	36.04	27.45
K K	3.03	1.66
Ca K	0.64	0.34
Ti K	0.36	0.16
Fe K	3.17	1.21
Totals	100.00	

Quantitative results

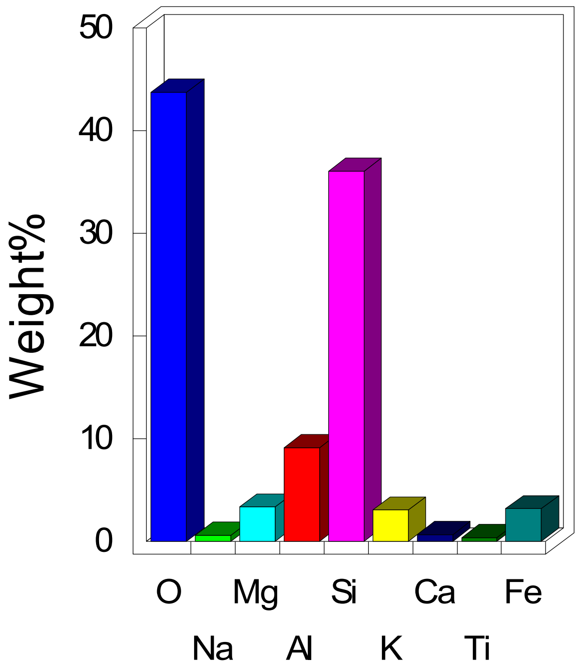

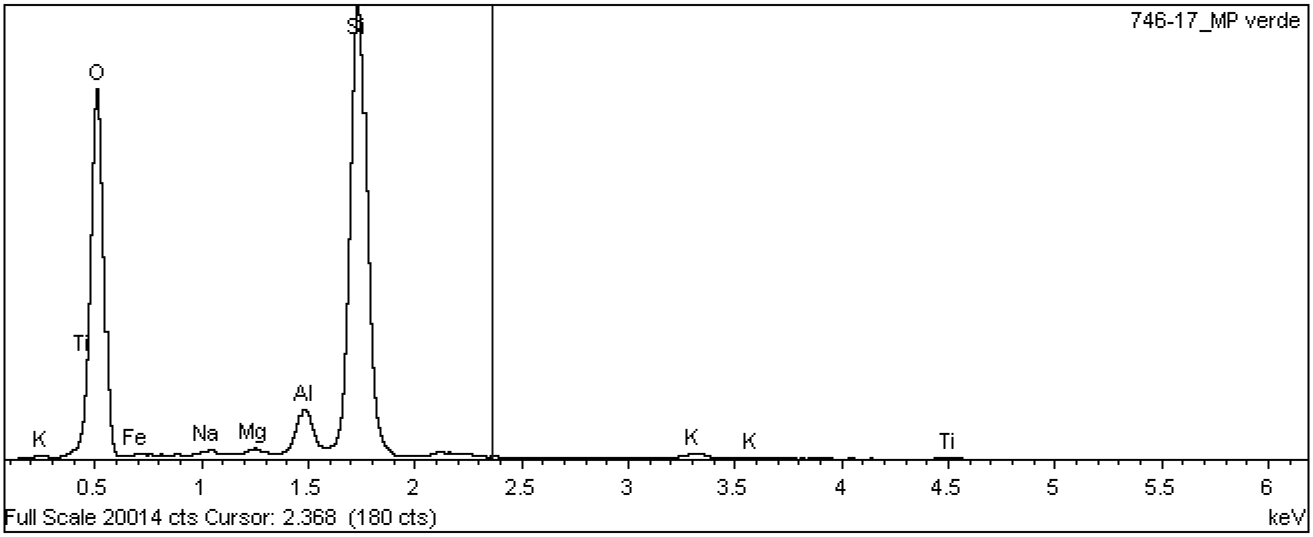

Spectrum processing :
No peaks omitted

Processing option : All elements analyzed (Normalised)
Number of iterations = 4

Standard :
O SiO2 1-Jun-1999 12:00 AM
Na Albite 1-Jun-1999 12:00 AM
Mg MgO 1-Jun-1999 12:00 AM
Al Al2O3 1-Jun-1999 12:00 AM
Si SiO2 1-Jun-1999 12:00 AM
K MAD-10 Feldspar 1-Jun-1999 12:00 AM
Ti Ti 1-Jun-1999 12:00 AM
Fe Fe 1-Jun-1999 12:00 AM

Element	Weight%	Atomic%
O K	67.56	78.67
Na K	0.38	0.31
Mg K	0.36	0.28
Al K	2.50	1.73
Si K	27.85	18.48
K K	0.55	0.26
Ti K	0.30	0.11
Fe K	0.50	0.17
Totals	100.00	

Quantitative results

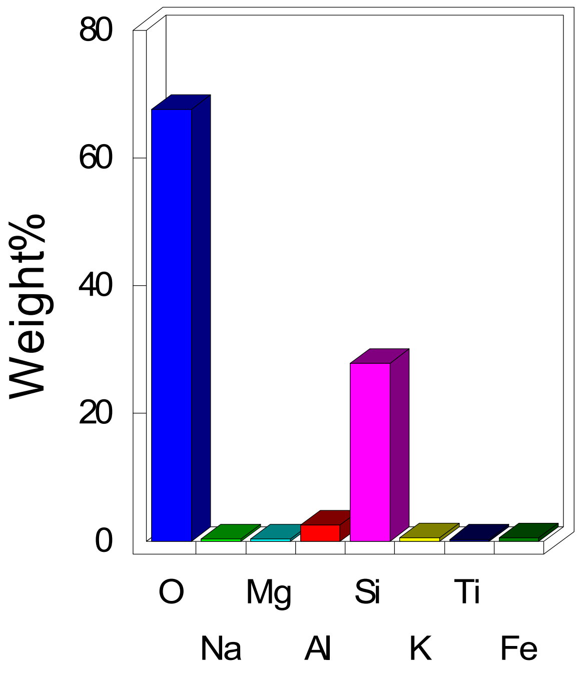

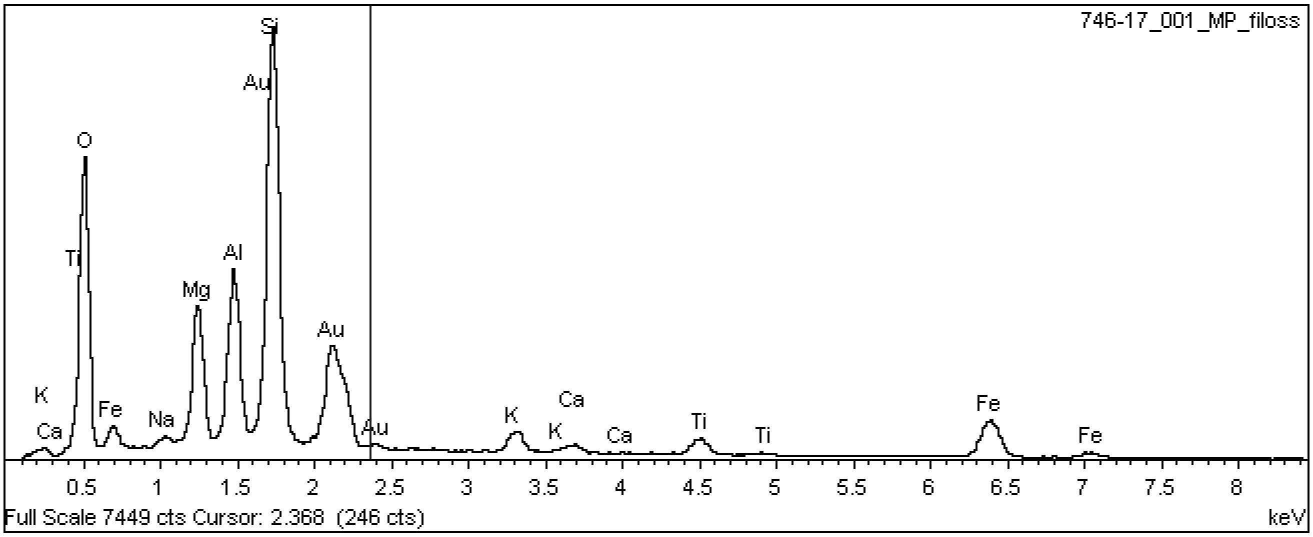

Spectrum processing :
No peaks omitted

Processing option : All elements analyzed (Normalised)
Number of iterations = 3

Standard :
O SiO2 1-Jun-1999 12:00 AM
Na Albite 1-Jun-1999 12:00 AM
Mg MgO 1-Jun-1999 12:00 AM
Al Al2O3 1-Jun-1999 12:00 AM
Si SiO2 1-Jun-1999 12:00 AM
K MAD-10 Feldspar 1-Jun-1999 12:00 AM
Ca Wollastonite 1-Jun-1999 12:00 AM
Ti Ti 1-Jun-1999 12:00 AM
Fe Fe 1-Jun-1999 12:00 AM

Quantitative results

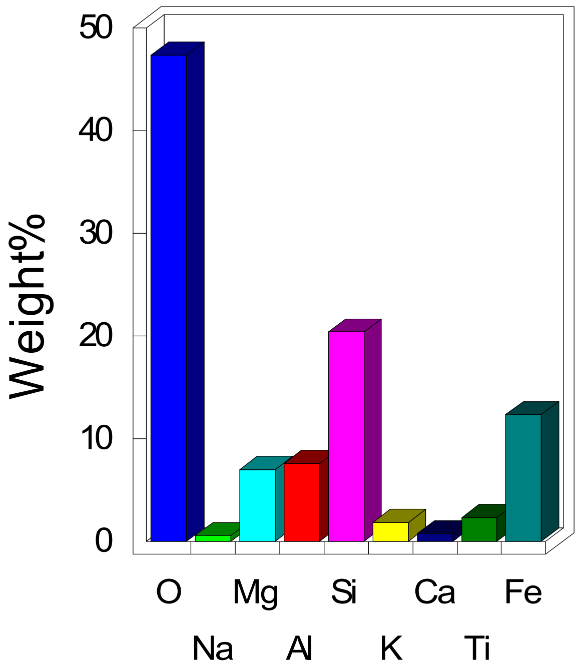

Element	Weight%	Atomic%
O K	47.33	64.18
Na K	0.56	0.53
Mg K	6.98	6.23
Al K	7.56	6.08
Si K	20.38	15.74
K K	1.83	1.01
Ca K	0.72	0.39
Ti K	2.30	1.04
Fe K	12.34	4.79
Totals	100.00	

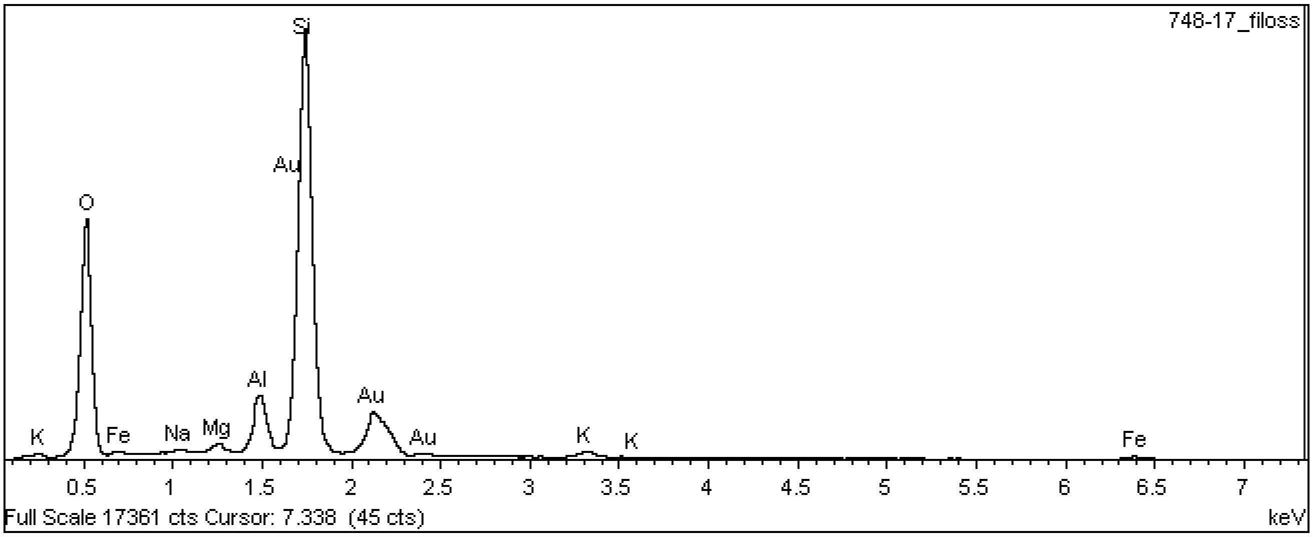

Spectrum processing :
No peaks omitted

Processing option : All elements analyzed (Normalised)
Number of iterations = 4

Standard :
O SiO2 1-Jun-1999 12:00 AM
Na Albite 1-Jun-1999 12:00 AM
Mg MgO 1-Jun-1999 12:00 AM
Al Al2O3 1-Jun-1999 12:00 AM
Si SiO2 1-Jun-1999 12:00 AM
K MAD-10 Feldspar 1-Jun-1999 12:00 AM
Fe Fe 1-Jun-1999 12:00 AM

Quantitative results

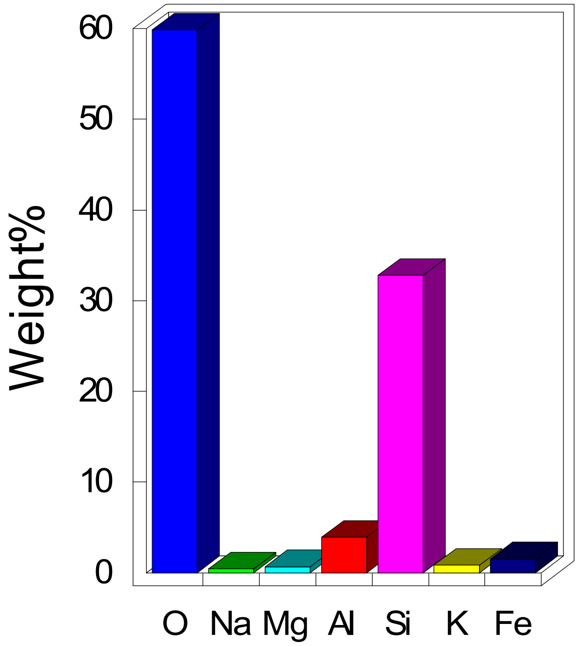

Element	Weight%	Atomic%
O K	59.84	72.66
Na K	0.42	0.35
Mg K	0.66	0.53
Al K	3.91	2.82
Si K	32.79	22.68
K K	0.84	0.42
Fe K	1.55	0.54
Totals	100.00	

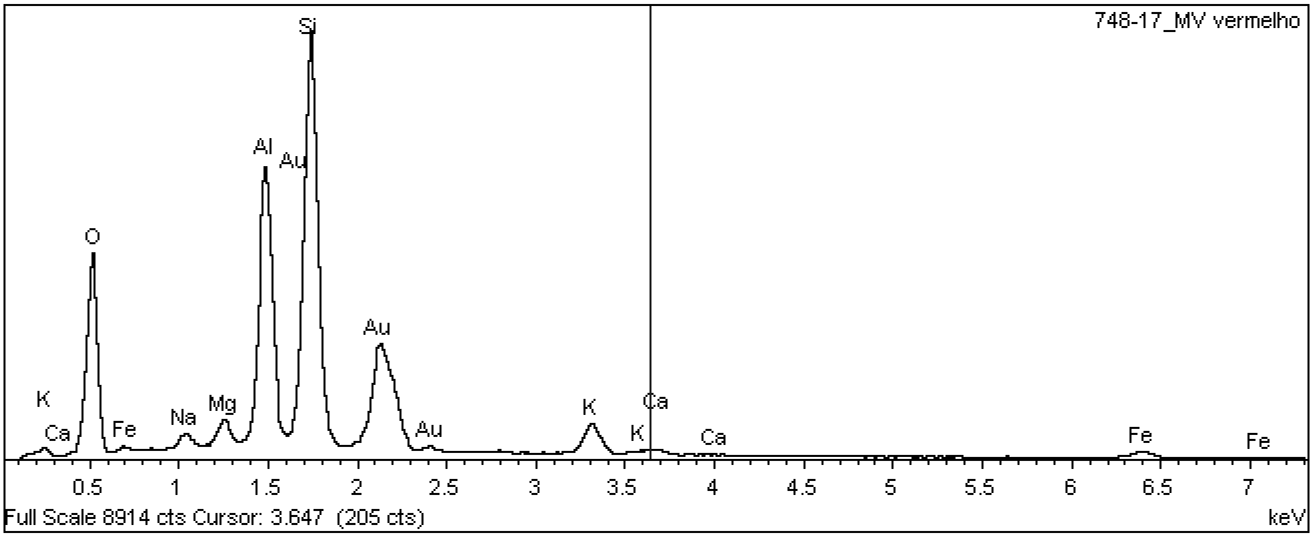

Spectrum processing :
No peaks omitted

Processing option : All elements analyzed (Normalised)
Number of iterations = 4

Standard :
O SiO2 1-Jun-1999 12:00 AM
Na Albite 1-Jun-1999 12:00 AM
Mg MgO 1-Jun-1999 12:00 AM
Al Al2O3 1-Jun-1999 12:00 AM
Si SiO2 1-Jun-1999 12:00 AM
K MAD-10 Feldspar 1-Jun-1999 12:00 AM
Ca Wollastonite 1-Jun-1999 12:00 AM
Fe Fe 1-Jun-1999 12:00 AM

Quantitative results

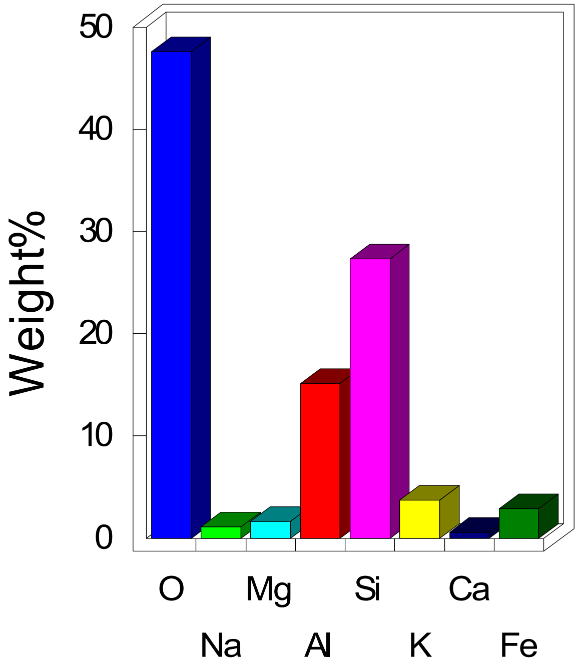

Element	Weight%	Atomic%
O K	47.57	62.14
Na K	1.11	1.01
Mg K	1.62	1.39
Al K	15.11	11.71
Si K	27.34	20.35
K K	3.75	2.00
Ca K	0.59	0.31
Fe K	2.91	1.09
Totals	100.00	

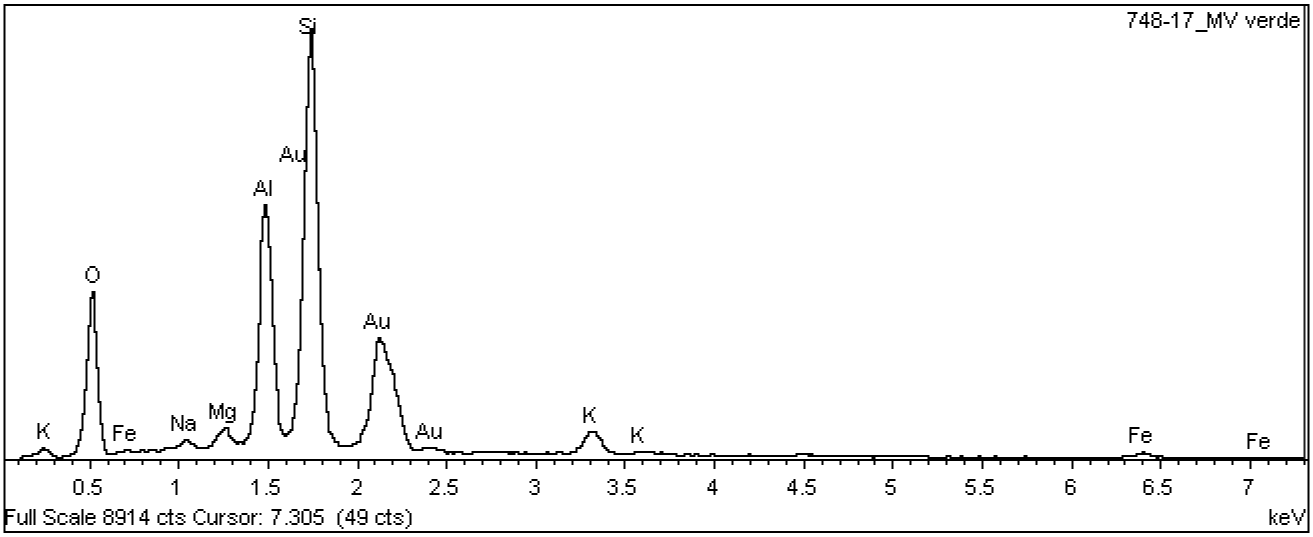

Spectrum processing :
Peak possibly omitted : 4.515 keV

Processing option : All elements analyzed (Normalised)
Number of iterations = 4

Standard :
O SiO2 1-Jun-1999 12:00 AM
Na Albite 1-Jun-1999 12:00 AM
Mg MgO 1-Jun-1999 12:00 AM
Al Al2O3 1-Jun-1999 12:00 AM
Si SiO2 1-Jun-1999 12:00 AM
K MAD-10 Feldspar 1-Jun-1999 12:00 AM
Fe Fe 1-Jun-1999 12:00 AM

Element	Weight%	Atomic%
O K	45.67	60.11
Na K	0.67	0.62
Mg K	1.26	1.10
Al K	15.01	11.72
Si K	31.76	23.81
K K	3.34	1.80
Fe K	2.28	0.86
Totals	100.00	

Quantitative results

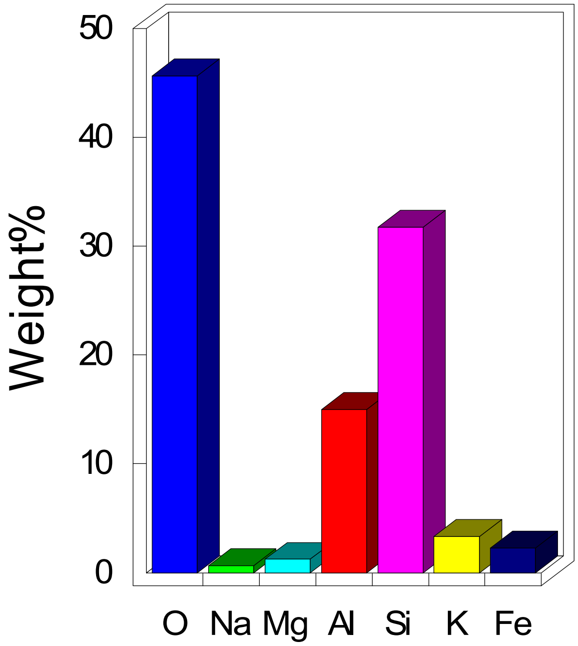

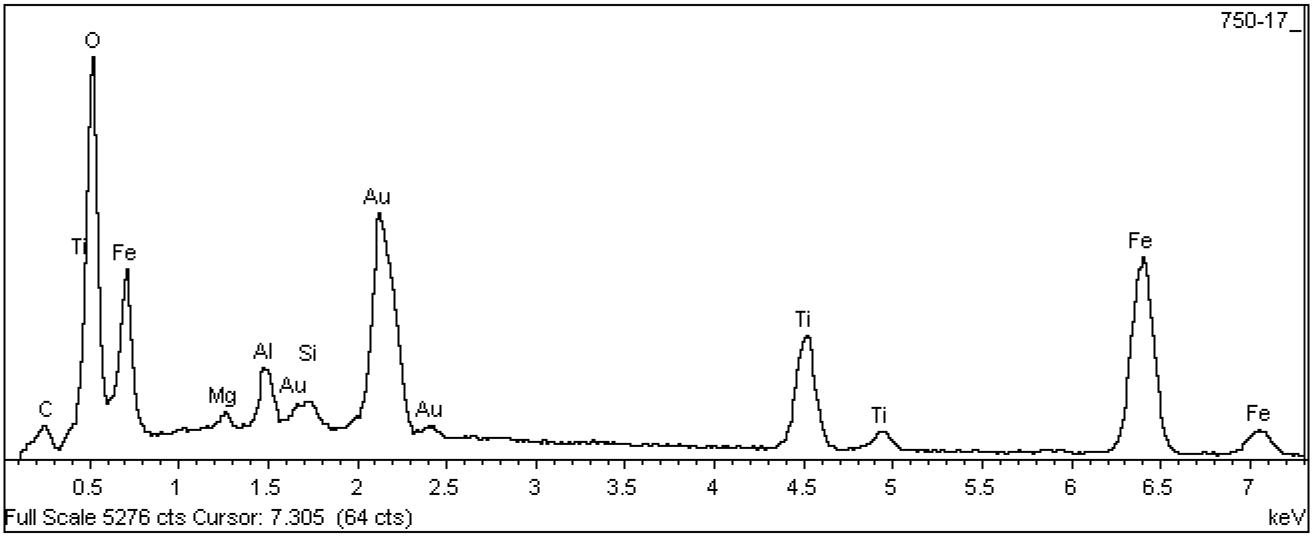

Spectrum processing :
No peaks omitted

Processing option : All elements analyzed (Normalised)
Number of iterations = 4

Standard :
C CaCO3 1-Jun-1999 12:00 AM
O SiO2 1-Jun-1999 12:00 AM
Mg MgO 1-Jun-1999 12:00 AM
Al Al2O3 1-Jun-1999 12:00 AM
Si SiO2 1-Jun-1999 12:00 AM
Ti Ti 1-Jun-1999 12:00 AM
Fe Fe 1-Jun-1999 12:00 AM

Quantitative results

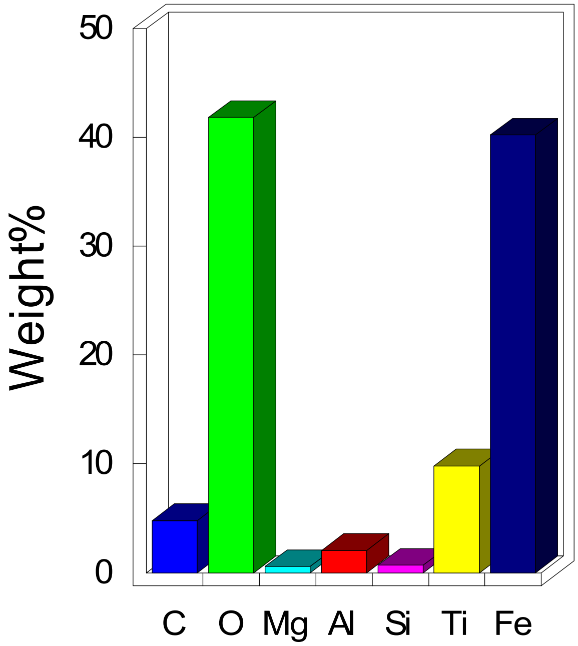

Element	Weight%	Atomic%
C K	4.80	9.84
O K	41.83	64.32
Mg K	0.59	0.60
Al K	2.06	1.87
Si K	0.69	0.60
Ti K	9.82	5.04
Fe K	40.22	17.72
Totals	100.00	
